# Baichuan Baile Formula, a Promising Herbal Dietary Supplement, Exerts Antidepressant‐Like Effects by Modulating the Serotoninergic System in Mouse Models

**DOI:** 10.1002/fsn3.70819

**Published:** 2025-09-03

**Authors:** Shuai‐Ming Zhu, Chun‐Xue Gao, Zi‐Jia Jin, Fu‐Yao Luo, Ting Feng, Jing‐Cao Li, Yu Yang, Rui Xu, Hao Ma, Chang‐Wei Li, Rui Xue, Jun‐Jie Shan, You‐Zhi Zhang

**Affiliations:** ^1^ Beijing Institute of Pharmacology and Toxicology Beijing China; ^2^ North China University of Science and Technology Tangshan China; ^3^ Beijing Technology and Business University Beijing China; ^4^ Nanjing University of Chinese Medicine Nanjing China

**Keywords:** antidepressant‐like, Baichuan Baile, functional foods, network pharmacology, serotoninergic

## Abstract

Baichuan Baile (BCBL) is a novel formula that incorporates three herbs and a single food additive, all of which are widely used in ordinary or functional foods. It is a promising dietary supplement for the treatment of depression based on TCM principles and nutritional assessments. Our study was designed to explore BCBL's antidepressant‐like effects and elucidate its underlying mechanisms. First, the antidepressant‐like effects of BCBL were predicted through network pharmacology and validated using systematic depression models. Next, the 5‐HTP‐induced head‐twitch test, yohimbine‐induced death test, and reserpine‐induced ptosis, hypothermia, and akinesia test were conducted to assess BCBL's impact on monoaminergic function. Additionally, the effects of BCBL on monoamine neurotransmitters were measured using HPLC‐ECD and ELISA. As a result, an integrated analysis using the TCMSP and Genecards databases identified 37 active components sharing 192 targets associated with depression. Further construction of the PPI network, along with GO and KEGG enrichment analyses, revealed a comprehensive and synergistic interaction between BCBL and depression, also predicting that the serotoninergic system might partially mediate the antidepressant‐like effects of BCBL. Subsequent pharmacological tests indicated that BCBL demonstrated significant antidepressant‐like effects on behavioral despair mice, CRS and CNE‐induced depression‐like mice, and chronic reserpine‐induced depression‐like mice. The DDI tests, HPLC‐ECD, and ELISA analyses validated that BCBL's antidepressant‐like effects were mediated through upregulation of the serotoninergic system. Our findings provided a safe and effective candidate for the amelioration of depression that deserves further investigations and explorations.

## Graphical abstract

BCBL is a promising dietary supplement for the treatment of depression. Its potential in treating depression was predicted by network pharmacology and verified through systematic experiments. BCBL warrants further investigations and explorations for its safety and efficacy.

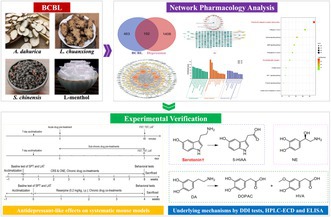

**Abbreviations**
5‐HIAA: 5‐hydroxyindole‐3‐acetic acid
5‐HTP: 5‐hydroxytryptophan

*A. dahurica*

: *Angelica dahurica* (Fisch. ex Hoffm.) Benth. et Hook. f.
ANOVA: analysis of variance
BCBL: Baichuan Baile
BP: biological processes
BSA: bovine serum albumin
CC: cellular component
CNE: chronic noise exposure
CRS: chronic restraint stress
DA: dopamine
DAergic: dopaminergic
DDI: drug–drug interaction
DL: drug‐likeness
DMSO: dimethyl sulfoxide
DOPAC: 3,4‐dihydroxyphenylacetic acid
ELISA: enzyme‐linked immunosorbent assay
FST: forced swimming test
GO: Gene Ontology
HPLC‐ECD: high‐performance liquid chromatography with electrochemical detection
HVA: homovanillic acid
KEGG: Kyoto Encyclopedia of Genes and Genomes
*L. chuanxiong*
: *Ligusticum chuanxiong* Hort.
LAT: locomotor activity test
MF: molecular function
NE: norepinephrine
NEergic: norepinephrinergic
OB: oral bioavailability
PFC: prefrontal cortex
PPI: protein–protein interaction

*S. chinensis*

: *Schisandra chinensis* (Turcz.) Baill.
SD: standard deviation
SPT: sucrose preference test
TCM: traditional Chinese medicine
TST: tail suspension test

## 1 Introduction

As reported by the World Health Organization, over 340 million people are suffering from depression, and the disease burden is projected to rank first by 2030 (Hong et al. 2024; Zhu et al. 2024). So far, pharmacotherapy is still the main treatment for depression, supplemented by phytotherapy, psychotherapy, and physiotherapy in clinical practice (Du et al. 2024). Over the past three decades, significant progress has been made in the development of antidepressants, and the commonly used clinical antidepressants are still based on the hypothesis of monoamine neurotransmitter depletion. According to the chemical structures and mechanisms of action, they are divided into selective serotonin reuptake inhibitors, selective norepinephrine (NE) reuptake inhibitors, serotonin and NE reuptake inhibitors, monoamine oxidase inhibitors, and tricyclic antidepressants (Wu et al. 2025; Zhu et al. 2023). However, it usually costs several weeks or even months to achieve ideal clinical effects with the above chemical antidepressants, followed by several toxic side effects, such as suicidal tendencies, sexual dysfunction, and sleep disorders (Lu et al. 2024; Zhong et al. 2024). Therefore, it is of great value to develop complementary and alternative therapies from herbal dietary supplements (Luo et al. 2024).

In the theory of traditional Chinese medicine (TCM), the primary pathogenesis of depression is closely related to the imbalance of Yin and Yang, as well as the stagnation of Qi and blood. The treatment of depression usually involves nourishing Yin and Jin, dispersing stagnated Qi and blood, soothing the liver, and strengthening the spleen (Wu et al. 2025; Zhong et al. 2024). Baichuan Baile (BCBL) is a novel formula based on TCM principles and nutritional assessments, comprising *Angelica dahurica* (Fisch. ex Hoffm.) Benth. et Hook. f. ( *A. dahurica* ), *Ligusticum chuanxiong* Hort. (*L. chuanxiong*), *Schisandra chinensis* (Turcz.) Baill. ( *S. chinensis* ), and L‐menthol. According to TCM theories, *A. dahurica* is known for dispelling wind and reducing swelling, *L. chuanxiong* is commonly used to soothe the liver and disperse stagnated blood, *S. chinensis* is characterized by tonifying Qi and nourishing Jin, and L‐menthol is effective at refreshing the mind and inducing resuscitation (Chinese Pharmacopoeia Commission 2019). Modern pharmacological studies also demonstrated that active fractions or monomer components from herbs of BCBL exerted effective antidepressant‐like and neuroprotective effects in several animal models (Ran et al. 2011; Wang et al. 2019; Yang et al. 2022; Zhao et al. 2022). As defined by the National Health Commission of the People's Republic of China, *A. dahurica* serves dual roles as both food and medicine, *L. chuanxiong* and *S. chinensis* are commonly used in functional foods, and L‐menthol is classified as a non‐toxic food additive. Therefore, BCBL is a safe and promising functional food for ameliorating depression, which necessitates systematic explorations.

In our study, the potential antidepressant‐like effects and underlying mechanisms of BCBL were first predicted by network pharmacology analysis (Chen et al. 2024). Next, the forced swimming test (FST), the tail suspension test (TST), and the locomotor activity test (LAT) in mice were conducted to rapidly validate the antidepressant‐like effects of both acute and sub‐chronic pre‐treatments with BCBL (Wang et al. 2016; Zhu et al. 2023). Subsequently, two depression models, chronic restraint stress (CRS) and chronic noise exposure (CNE)‐induced and chronic reserpine‐induced depression‐like mice, were established based on prior studies to elucidate the antidepressant‐like effects of chronic BCBL co‐treatments in mice (Abdel‐Rasoul et al. 2023; Cheng et al. 2022; Qian et al. 2023; Qiao et al. 2016). To determine the potential mechanisms of BCBL, 5‐hydroxytryptophan (5‐HTP)‐induced head‐twitch test, yohimbine‐induced death test, and reserpine‐induced ptosis, hypothermia, and akinesia test were conducted to explore the effects of BCBL on the monoaminergic function in mice (Xu et al. 2017; Zhu et al. 2023). In parallel, high‐performance liquid chromatography with electrochemical detection (HPLC‐ECD) and enzyme‐linked immunosorbent assay (ELISA) were further performed to accurately determine the effects of BCBL on the monoamine neurotransmitter levels (Zhang et al. 2012).

## 2 Materials and Methods

### 2.1 Extraction and Content Analysis of BCBL Water Extract

#### 2.1.1 Plant Materials

Dried roots of *A. dahurica* and *L. chuanxiong*, dried fruits of *S. chinensis* , and L‐menthol were obtained from Beijing Tongrentang Co. Ltd. (Beijing, China). The Chinese names, batch numbers, dosages, and origins were described in Table 1. It is worth noting that BCBL has been granted a national invention patent in China, with the authorization number CN116570659B.

**TABLE 1:** Detailed description of BCBL.

Name	Chinese name	Batch number	Dosage (g)	Origin
*A. dahurica*	Bai Zhi	201116	5–20	Henan Province
*L. chuanxiong*	Chuan Xiong	B8031511	3–12	Sichuan Province
*S. chinensis*	Wu Wei Zi	21041002	3–12	Liaoning Province
L‐menthol	Bo He Nao	210307025	0.02–0.12	Guangdong Province

*Note:* The prescribed dosages specify the therapeutic amounts of herbs and food additives to be taken by an adult each day.

#### 2.1.2 Extraction

The dried materials of *A. dahurica* , *L. chuanxiong*, and *S. chinensis* were mixed in specific ratios of 5:3:3 and soaked in distilled water at tenfold their weight for a duration of 60 min. The herbal‐water mixture was then brought to a boil and simmered for 60 min. The supernatant of the mixture was filtered, and the remaining herbal marc was further boiled with tenfold distilled water for another 60 min. Subsequently, the supernatants were combined, centrifuged, concentrated, and dried at 80°C to obtain the water extract of BCBL. Before drug administration, BCBL water extract was compounded with L‐menthol in the specific ratios as described in Table 1.

#### 2.1.3 Content Analysis

The contents of total coumarins and phenolic acids were determined using the reported colorimetric methods, with imperatorin and ferulic acid as the standards, respectively (Li et al. 2007; Shi et al. 2015). The phenol‐sulfuric acid assay was conducted to assess the content of total carbohydrates in BCBL water extract, using glucose as the standard (Yu et al. 2017). The Bradford's assay was employed to determine the protein concentration, with bovine serum albumin (BSA) serving as the standard (Zou et al. 2023). The standards for imperatorin (B20929) and ferulic acid (B20007) were obtained from Shanghai Yuanye Co. Ltd. (Shanghai, China). The standards for glucose (D9434) and BSA (A1933) were acquired from Sigma‐Aldrich in San Francisco, California.

### 2.2 Network Pharmacology Analysis

#### 2.2.1 Screening of Active Compounds From BCBL

The active constituents of *A. dahurica* , *L. chuanxiong*, and *S. chinensis* were obtained from the TCMSP database (http://tcmspw.com/tcmsp.php). Oral bioavailability (OB) of at least 30% and drug‐likeness (DL) of at least 0.18 were the criteria used to select active compounds from BCBL. Although the DL of L‐menthol is less than 0.18, it was reasonably selected as an active constituent of BCBL for further network pharmacology analysis, due to its status as a safe and non‐toxic monoterpenoid with various pharmacological activities, such as neuroprotection, antidepressant‐like effects, and penetration enhancement (Du et al. 2020; Wang et al. 2019).

#### 2.2.2 Prediction of BCBL‐Related and Depression‐Related Targets

The chemical structures of active compounds in BCBL, in SDF file format, were acquired from the PubChem database (https://pubchem.ncbi.nlm.nih.gov/). Subsequently, these SDF files were imported into the Swiss Target Prediction platform (http://www.swisstargetprediction.ch/) to predict the potential targets of BCBL, setting the species as “ *Homo sapiens* ” and using the reversed pharmacophore matching approach. In parallel, the depression‐related targets were identified through the Genecards database (https://www.genecards.org/) (Chen et al. 2024; Wu et al. 2025).

#### 2.2.3 Network Construction of Active Compounds and Depression‐Related Targets

Using the R programming language package, we generated a Venn diagram to visualize the overlap between BCBL‐related and depression‐related targets (Jia et al. 2021). Subsequently, the Cytoscape 3.7.2 software was used to create a visual network illustrating the interactions between active compounds of BCBL and overlapping depression‐related targets. This network provided insights into the potential mechanisms of BCBL's action against depression (Doncheva et al. 2019).

#### 2.2.4 Protein–Protein Interaction (PPI) Network Analysis

The potential BCBL targets for treating depression were uploaded to the STRING database (https://www.string‐db.org/) to construct a PPI network. The analysis was limited to “ *Homo sapiens* ” with the interaction confidence threshold set to “highest confidence” > 0.7 to ensure data reliability, using default settings for other parameters. Subsequently, the Cytoscape 3.7.2 software was used to identify the core network genes, focusing on the most central and potentially influential ones (Szklarczyk et al. 2023).

#### 2.2.5 Gene Ontology (GO) and Kyoto Encyclopedia of Genes and Genomes (KEGG) Enrichment Analysis

GO assays were conducted through the DAVID database (https://david.ncifcrf.gov/) to identify potential target genes across biological processes (BP), cellular components (CC), and molecular functions (MF). Simultaneously, KEGG enrichment analysis was performed to elucidate key signaling pathways related to these target genes. To delve into the functional implications and pathway interactions of these proteins with BCBL targets in depression, the R Software was employed for both GO and KEGG enrichment analyses. This comprehensive approach aided in visualizing and understanding the potential mechanisms by which BCBL may alleviate depression (Kanehisa et al. 2017).

### 2.3 Antidepressant‐Like Effects of Acute Pre‐Treatment With BCBL in Behavioral Despair Mice

#### 2.3.1 Animals and Grouping

One hundred and fifty male ICR mice, weighing 20 ± 2 g, were sourced from SPF Biological Technology Co. Ltd. (Beijing, China). These mice were subsequently allocated into five groups of 30 each: the control group (Vehicle; 20 mL/kg, i.g.), the duloxetine hydrochloride group (DLX; 20 mg/kg, i.g.), and a series of doses of BCBL groups (BCBL water extract & L‐menthol; 300 mg/kg & 1.625 mg/kg, 600 mg/kg & 3.25 mg/kg, and 1200 mg/kg & 6.5 mg/kg, i.g.). They were housed in a controlled environment with a 12‐h light–dark cycle at 24°C ± 1°C and allowed to acclimate for 7 days before experimentation. Water and food were provided ad libitum unless specified otherwise. The experiment was conducted ethically, following the approval of the Institutional Animal Care and Use Committee (Ethics approval number: IACUC‐DWZX‐2021‐618), with measures in place to minimize the mice's suffering.

#### 2.3.2 Drug Treatment

After a 7‐day environmental adaptation period, oral administration of DLX, sourced from Shanghai Wandai Pharmaceutical Co. Ltd. (Shanghai, China), and BCBL was initiated. The DLX dosage was selected based on prior research (Zhu et al. 2023), while BCBL doses were determined using clinical herb dosage, extraction yield, and body surface area‐based equivalent dose calculations between human and mouse (Chinese Pharmacopoeia Commission 2019; Zhao and Sun 2010). Notably, DLX was dissolved in 1% dimethyl sulfoxide (DMSO) vehicle. L‐menthol was first dissolved in the same 1% DMSO vehicle before being compounded with BCBL water extract at the specified doses. The control group received an equivalent volume of the vehicle orally.

#### 2.3.3 Behavioral Testing

Sixty minutes after the drug treatment, 10 mice from each group were tested for the antidepressant‐like effects of BCBL using the FST and TST, which assessed the display of despair in response to an inescapable situation. The remaining 10 mice from each group were subjected to the LAT to evaluate BCBL's influence on locomotor activity. The procedures for the FST, TST, and LAT followed our previously published methods (Zhu et al. 2023).

### 2.4 Antidepressant‐Like Effects of Sub‐Chronic Pre‐Treatments With BCBL in Behavioral Despair Mice

#### 2.4.1 Animals and Grouping

One hundred and fifty male ICR mice, weighing 20 ± 2 g, were acquired from the same supplier. These mice were subsequently allocated into five groups of 30 each: the control group (Vehicle; 20 mL/kg, i.g.), the DLX group (DLX; 20 mg/kg, i.g.), and a series of doses of BCBL groups (BCBL water extract & L‐menthol; 300 mg/kg & 1.625 mg/kg, 600 mg/kg & 3.25 mg/kg, and 1200 mg/kg & 6.5 mg/kg, i.g.). They were then kept and cared for in an ethical manner, as previously described.

#### 2.4.2 Drug Treatment

After a 7‐day environmental adaptation period, the drug was dispensed as previously mentioned and delivered orally for 7 days. The control group received an equivalent volume of the vehicle orally.

#### 2.4.3 Behavioral Testing

The FST, TST, and LAT were conducted to evaluate the effects of sub‐chronic pre‐treatments with BCBL in behavioral despair mice, 60 min after the drug treatment on day 7. The experimental procedures followed those previously described.

### 2.5 Antidepressant‐Like Effects of Chronic Co‐Treatments With BCBL in CRS and CNE‐Induced Depression‐Like Mice

#### 2.5.1 Animals and Grouping

Male ICR mice, another 50 in number and weighing 20 ± 2 g, were sourced from the same supplier and divided into five groups at random, including the control group (Sham+Vehicle; 20 mL/kg, i.g.), the model group (CRS & CNE+Vehicle; 20 mL/kg, i.g.), the DLX group (CRS & CNE + DLX; 20 mg/kg, i.g.), and a series of doses of BCBL groups (CRS & CNE+BCBL water extract & L‐menthol; 1200 mg/kg & 3.25 mg/kg, and 2400 mg/kg & 6.5 mg/kg, i.g.), with 10 animals in each group. They were then kept and cared for ethically, as previously described.

#### 2.5.2 Establishment of CRS and CNE‐Induced Depression‐Like Mice and Drug Treatment

After a 7‐day environmental adaptation, the control group was exempt from the CRS and CNE procedure, had normal access to water and food, and received an equivalent volume of the vehicle orally. The model group underwent 6 h of CRS during the day and 12 h of CNE at night (Cheng et al. 2022; Qian et al. 2023; Qiao et al. 2016), also receiving an equivalent volume of the vehicle orally. All other groups experienced the same stress stimuli as the model group and were administered different drugs at the aforementioned doses for a continuous 8‐week period. Notably, one mouse from the model group and two mice each from the positive group and both low and high dose BCBL groups died unexpectedly during the initial CRS procedure.

#### 2.5.3 Behavioral Testing

The sucrose preference test (SPT) was undertaken on day‐2 and day 56 to assess the sucrose preference index in mice before the CRS and CNE procedure and after the combined CRS and CNE with chronic drug co‐treatments, respectively. In summary, a 48‐h sucrose preference training phase was initiated. Following this, mice were given access to both distilled water and a 1% sucrose solution, with the bottle positions switched after 12 h. The sucrose preference index was calculated for the subsequent 24 h using the formula: Sucrose preference index (%) = (Daily consuming amount of sucrose solution/Daily consuming amount of water and sucrose solution) × 100% (Wang et al. 2016).

The LAT was conducted on day‐1 and day 57 using the previously mentioned method to assess mice's locomotor activity before the CRS & CNE procedure and after the combined CRS & CNE with chronic drug co‐treatments, respectivel. Additionally, the distance rate in the central area within the first 10 min was calculated to evaluate the anxiety levels of CRS & CNE‐induced mice during LAT. The formula for this calculation was: Rate of distance in the center area (%) = (Distance in the center area/Total distance) × 100% (Zhu et al. 2024).

The TST and FST were conducted on days 58 and 59 to assess the impact of chronic BCBL co‐treatments on the immobility time of CRS and CNE‐induced depression‐like mice. The experimental procedures followed those previously described (Zhu et al. 2023).

### 2.6 Antidepressant‐Like Effects of Chronic Co‐Treatments With BCBL in Chronic Reserpine‐Induced Depression‐Like Mice

#### 2.6.1 Animals and Grouping

Another 78 male ICR mice, weighing 20 ± 2 g, were randomly assigned to six groups from the same supplier: the control group (Vehicle+Vehicle; 20 mL/kg, i.g.), the model group (Reserpine+Vehicle; 20 mL/kg, i.g.), the DLX group (Reserpine+DLX; 20 mg/kg, i.g.), and a series of doses of BCBL groups (Reserpine+BCBL water extract & L‐menthol; 600 mg/kg & 1.625 mg/kg, 1200 mg/kg & 3.25 mg/kg, and 2400 mg/kg & 6.5 mg/kg, i.g.). Each group consisted of 13 mice. The mice were housed and cared for ethically, as detailed in previous descriptions.

#### 2.6.2 Establishment of Chronic Reserpine‐Induced Depression‐Like Mice and Drug Treatment

Following a 7‐day environmental adaptation, DLX and varying doses of BCBL were orally administered as previously described. Concurrently, both the control and model groups received an equivalent volume of the vehicle orally. Subsequently, reserpine was intraperitoneally injected at a dose of 0.2 mg/kg, dissolved in a sterile, endotoxin‐free 0.2% acetic acid solution, 60 min after the oral treatments. The control group also received an equivalent volume of the vehicle intraperitoneally. This regimen of drug treatment and reserpine injections was maintained continuously for 4 weeks (Abdel‐Rasoul et al. 2023). Notably, one mouse from each of the model, positive, and low and high dose BCBL groups, as well as two mice from the middle dose BCBL group, died during the establishment of a chronic reserpine‐induced depression‐like model.

#### 2.6.3 Behavioral Testing

The SPT was administered on day‐2 and day 28 to assess the sucrose preference index in mice before and after drug co‐treatments and reserpine injections. The LAT was conducted on day‐1 and day 29 to evaluate the locomotor activity in mice under the same conditions. Additionally, the TST and FST were conducted on day 30 and day 31 to detect the effects of chronic BCBL co‐treatments on the immobility time in chronic reserpine‐induced depression‐like mice. All experimental procedures followed those previously described.

#### 2.6.4 Sample Collection

After completing the behavioral tests in chronic reserpine‐induced depression‐like mice, serum was extracted from the retro‐orbital sinus. Subsequently, all mice were euthanized by cervical dislocation to collect the hippocampus and prefrontal cortex (PFC). These brain regions were then analyzed to determine the levels of serotonin and NE by ELISA.

### 2.7 Effects of BCBL on the Monoaminergic Systems by Drug–Drug Interaction (DDI) Tests

#### 2.7.1 Effects of BCBL on the Serotoninergic System by 5‐HTP‐Induced Head‐Twitch Test in Mice

Another 50 male ICR mice, weighing 20 ± 2 g, were randomly divided into five groups: the control group (5‐HTP+Vehicle; 20 mL/kg, i.g.), the DLX group (5‐HTP+DLX; Day 1–8, 20 mg/kg; Day 9, 5 mg/kg, i.g.), and a series of doses of BCBL groups (5‐HTP+BCBL water extract & L‐menthol; 300 mg/kg & 1.625 mg/kg, 600 mg/kg & 3.25 mg/kg, and 1200 mg/kg & 6.5 mg/kg, i.g.), with 10 animals in each group. They were then kept and cared for ethically, as previously described.

Following a 7‐day environmental adaptation, DLX and varying doses of BCBL were administered orally every day for 9 consecutive days. The control group received an equal volume of the vehicle orally. On day 9, 60 min after the drug administration, each mouse was given an intraperitoneal injection of 5‐HTP (120.0 mg/kg) and promptly placed in a separate plastic cage. After a 5‐min acclimation period, the total number of head twitches was recorded and analyzed over the subsequent 15 min (Corne et al. 1963).

#### 2.7.2 Effects of BCBL on the Norepinephrinergic (NEergic) System by Yohimbine‐Induced Death Test in Mice

Another 50 male ICR mice, weighing 20 ± 2 g, were randomly divided into five groups: the control group (Yohimbine+Vehicle; 20 mL/kg, i.g.), the DLX group (Yohimbine+DLX; 20 mg/kg, i.g.), and a series of doses of BCBL groups (Yohimbine+BCBL water extract & L‐menthol; 300 mg/kg & 1.625 mg/kg, 600 mg/kg & 3.25 mg/kg, and 1200 mg/kg & 6.5 mg/kg, i.g.), with 10 animals in each group. They were then kept and cared for ethically, as previously described.

Following a 7‐day environmental adaptation period, DLX and various doses of BCBL were orally administered for 9 consecutive days, whereas the control group received an equal volume of the vehicle orally. 60 min after the final drug treatment, each mouse was given an intraperitoneal injection of yohimbine (30 mg/kg). The mortality rate for each group was monitored and recorded within a 24‐h period (Xu et al. 2017).

#### 2.7.3 Effects of BCBL on the Serotoninergic, NEergic, and Dopaminergic (DAergic) Systems by Reserpine‐Induced Ptosis, Hypothermia, and Akinesia Test in Mice

Another 60 male ICR mice, weighing 20 ± 2 g, were randomly divided into six groups: the control group (Vehicle+Vehicle; 20 mL/kg, i.g.), the model group (Reserpine+Vehicle; 20 mL/kg, i.g.), the DLX group (Reserpine+DLX; 20 mg/kg, i.g.), and a series of doses of BCBL groups (Reserpine+BCBL water extract & L‐menthol; 300 mg/kg & 1.625 mg/kg, 600 mg/kg & 3.25 mg/kg, and 1200 mg/kg & 6.5 mg/kg, i.g.), with 10 animals in each group. They were then kept and cared for ethically, as previously described.

Following a 7‐day environmental adaptation period, DLX and various doses of BCBL were orally administered daily for 9 consecutive days. Both the control and model groups received an equivalent volume of the vehicle orally. On day 9, 60 min after the drug treatment, reserpine was intraperitoneally injected at a dose of 2.5 mg/kg, dissolved in a sterile, endotoxin‐free 0.2% acetic acid solution. The control group also received an equivalent volume of the vehicle intraperitoneally. Each animal was then placed on a shelf located 20 cm above a table. The degrees of ptosis, hypothermia, and akinesia were recorded using the method previously reported (Zhu et al. 2023). Subsequently, all mice were euthanized by cervical dislocation to collect the hippocampus and PFC. These brain regions were then analyzed to determine the levels of serotonin, NE, dopamine (DA), 5‐hydroxyindole‐3‐acetic acid (5‐HIAA), 3,4‐dihydroxyphenylacetic acid (DOPAC), and homovanillic acid (HVA) by HPLC‐ECD.

### 2.8 Assays for Serotonin, NE, DA, and Related Metabolites in the Hippocampus and PFC of Mice From Reserpine‐Induced Ptosis, Hypothermia, and Akinesia Test by HPLC‐ECD

The hippocampus and PFC tissues of mice from reserpine‐induced ptosis, hypothermia, and akinesia tests were used to determine the effects of BCBL on the levels of serotonin, NE, DA, 5‐HIAA, DOPAC, and HVA by HPLC‐ECD. All experimental procedures followed those previously reported (Zhang et al. 2012).

### 2.9 Assays for Serotonin and NE in the Hippocampus and PFC of Chronic Reserpine‐Induced Depression‐Like Mice by ELISA

The serotonin and NE levels in the hippocampus and PFC of chronic reserpine‐induced depression‐like mice were measured through ELISA. Nanjing Jiancheng Bioengineering Institute (Nanjing, China) supplied the serotonin (H104) and NE (H096) ELISA kits, which were utilized following the manufacturer's instructions.

### 2.10 Statistical Analysis

Statistical analyses for behavioral testing and biochemical assays were conducted through SPSS 16.0 software. Count data were analyzed by Fisher's exact test. Measurement data were presented as mean ± standard deviation (SD) and visualized using GraphPad Prism 6.01 Software. For comparing two groups, the Student's *t*‐test was applied. For three or more groups, a one‐way analysis of variance (ANOVA) or two‐way repeated‐measures ANOVA was performed, followed by the post hoc LSD test. A *p* value of less than 0.05 was considered to indicate statistical significance in the study.

## 3 Results

### 3.1 Extraction and Content Analysis of BCBL Water Extract

The water extraction yield of BCBL reached 41.1%. Preliminary colorimetric assays were conducted to evaluate the composition of BCBL water extract. As detailed in Table 2, the results revealed the presence of total coumarins, total phenolic acids, total carbohydrates, and total proteins, with relative contents of 5.73%, 11.21%, 31.93%, and 8.54%, respectively.

**TABLE 2:** Content analysis of BCBL water extract.

Sample	Content (w/w, %)			
	Total coumarins	Total phenolic acids	Total carbohydrates	Total proteins
BCBL water extract	5.73	11.21	31.93	8.54

### 3.2 Network Pharmacology Analysis

#### 3.2.1 Screening of Active Compounds From BCBL

As shown in Table 3, a comprehensive TCMSP database analysis identified 37 active compounds from BCBL. Among these, 21 compounds meeting the selection criteria of OB ≥ 30% and DL ≥ 0.18 were linked to *A. dahurica* , with key components including ethyl oleate, β‐sitosterol, and stigmasterol. From *L. chuanxiong*, 7 active components were identified, such as mandenol, myricanone, perlolyrine, and so on. Additionally, 8 compounds with conforming OB and DL values, such as longikaurin A, deoxyharringtonine, and schizandrer B, were related to *S. chinensis* . The monomer component L‐menthol, known for its diverse pharmacological activities, was also selected for further network pharmacology analysis.

**TABLE 3:** 37 active compounds from BCBL through the TCMSP database.

No.	Name	Formula	OB (%)	DL	TCMSP ID	Source
1	Sen‐byakangelicol	C_21_H_22_O_7_	58.00	0.61	MOL005807	*A. dahurica*
2	Isoimperatorin	C_16_H_14_O_4_	45.46	0.23	MOL001942	*A. dahurica*
3	Stigmasterol	C_29_H_48_O	43.83	0.76	MOL000449	*A. dahurica*
4	Prangenin	C_16_H_14_O_5_	43.60	0.29	MOL013430	*A. dahurica*
5	ZINC03860434	C_24_H_38_O_4_	43.59	0.35	MOL001749	*A. dahurica*
6	{5‐[2′(R)‐Hydroxy‐3′‐methyl‐3′‐butenyl‐oxy]furocoumarin}	C_16_H_14_O_5_	42.85	0.26	MOL005792	*A. dahurica*
7	Byakangelicol	C_17_H_16_O_6_	41.42	0.36	MOL005800	*A. dahurica*
8	Phellopterin	C_17_H_16_O_5_	40.19	0.28	MOL002644	*A. dahurica*
9	4‐[(2S)‐2,3‐dihydroxy‐3‐methylbutoxy]furo[3,2‐g]chromen‐7‐one	C_16_H_16_O_6_	39.99	0.29	MOL005806	*A. dahurica*
10	Methyl icosa‐11,14‐dienoate	C_21_H_38_O_2_	39.67	0.23	MOL007514	*A. dahurica*
11	CLR	C_27_H_46_O	37.87	0.68	MOL000953	*A. dahurica*
12	Propyleneglycol monoleate	C_21_H_40_O_3_	37.60	0.26	MOL005802	*A. dahurica*
13	Linolein, 2‐mono—	C_31_H_38_O_4_	37.28	0.30	MOL003791	*A. dahurica*
14	β‐Sitosterol	C_29_H_50_O	36.91	0.75	MOL000358	*A. dahurica*
15	Prangenidin	C_16_H_14_O_4_	36.31	0.22	MOL003588	*A. dahurica*
16	Neobyakangelico I	C_17_H_16_O_6_	36.18	0.31	MOL005789	*A. dahurica*
17	Alloisoimperatorin	C_16_H_14_O_4_	34.80	0.22	MOL001939	*A. dahurica*
18	Ammidin	C_16_H_14_O_4_	34.55	0.22	MOL001941	*A. dahurica*
19	Supraene	C_30_H_50_	33.55	0.42	MOL001506	*A. dahurica*
20	Cnidilin	C_17_H_16_O_5_	32.69	0.28	MOL001956	*A. dahurica*
21	Ethyl oleate	C_20_H_38_O_2_	32.40	0.19	MOL002883	*A. dahurica*
22	Facid	C_19_H_19_N_7_O_6_	68.96	0.71	MOL000433	*L. chuanxiong*
23	Perlolyrine	C_16_H_12_N_2_O_2_	65.95	0.27	MOL002140	*L. chuanxiong*
24	Senkyunone	C_22_H_30_O_2_	47.66	0.24	MOL002151	*L. chuanxiong*
25	Wallichilide	C_25_H_32_O_5_	42.31	0.71	MOL002157	*L. chuanxiong*
26	Mandenol	C_20_H_36_O_2_	42.00	0.19	MOL001494	*L. chuanxiong*
27	Myricanone	C_21_H_24_O_5_	40.60	0.51	MOL002135	*L. chuanxiong*
28	Sitosterol	C_29_H_50_O	36.91	0.75	MOL000359	*L. chuanxiong*
29	Longikaurin A	C_20_H_28_O_5_	47.72	0.53	MOL004624	*S. chinensis*
30	Wuweizisu C	C_22_H_24_O_6_	46.27	0.84	MOL008992	*S. chinensis*
31	Deoxyharringtonine	C_28_H_37_NO_8_	39.27	0.81	MOL005317	*S. chinensis*
32	Gomisin R	C_22_H_24_O_7_	34.84	0.86	MOL008978	*S. chinensis*
33	Gomisin G	C_30_H_32_O_9_	32.68	0.83	MOL008974	*S. chinensis*
34	Angeloylgomisin O	C_28_H_34_O_8_	31.97	0.85	MOL008956	*S. chinensis*
35	Schizandrer B	C_28_H_34_O_9_	30.71	0.83	MOL008957	*S. chinensis*
36	Gomisin A	C_23_H_28_O_7_	30.69	0.78	MOL008968	*S. chinensis*
37	L‐menthol	C_10_H_20_O	59.33	0.03	MOL007330	L‐menthol

*Note:* L‐menthol was selected for further network pharmacology analysis due to its diverse pharmacological activities.

#### 3.2.2 Network Construction of Active Compounds and Depression‐Related Targets

**FIGURE 1:**
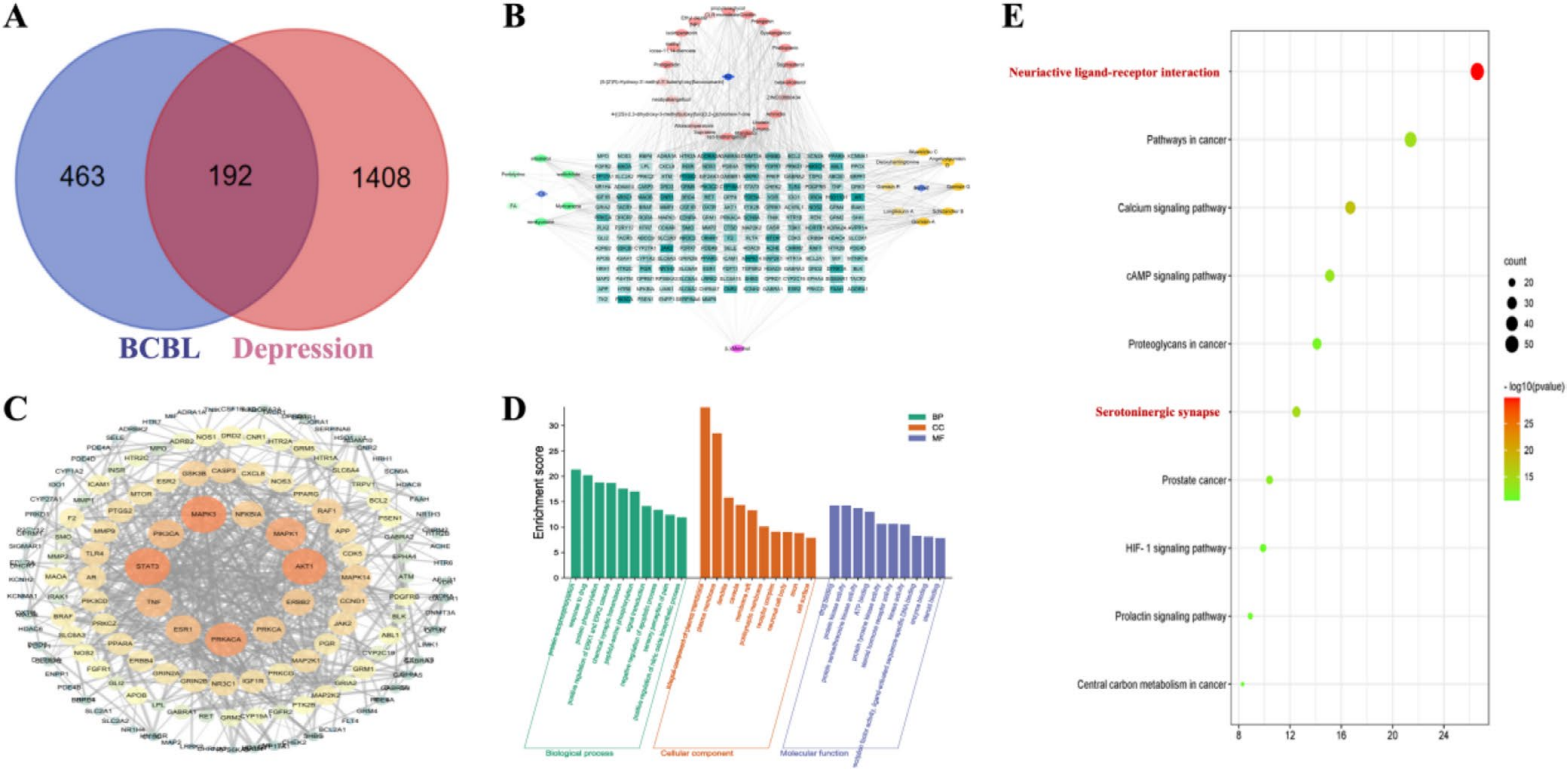
Network pharmacology analysis of BCBL in the treatment of depression. (A) Prediction of BCBL‐related and depression‐related targets. (B) Network construction of 37 active compounds and 192 overlapping targets. (C) PPI network analysis involving 192 overlapping targets. (D, E) GO and KEGG enrichment analysis.

The Swiss Target Prediction platform, utilizing a reversed pharmacophore matching approach, predicted 665 drug targets associated with BCBL. Meanwhile, a total of 1600 depression‐related targets were identified through the Genecards database. As depicted in Figure 1A, a Venn diagram comparison between the 665 BCBL‐related and 1600 depression‐related targets revealed 192 overlapping genes, which were selected for further analysis. Subsequently, the Cytoscape 3.7.2 software was used to construct a visual interaction network involving the 37 active compounds and the 192 overlapping targets (Figure 1B; Figure S1). The extensive target spectra associated with these compounds underscored the pivotal role of BCBL in regulating the biochemical pathways involved in depression. Moreover, it emphasized the therapeutic benefits of TCMs, characterized btheirts multi‐component and multi‐target approach.

#### 3.2.3 PPI Network Analysis

As illustrated in Figure 1C and Figure S2, the STRING database was employed to construct a PPI network involving the 192 overlapping targets. Using Cytoscape 3.7.2 software, the core network genes were systematically identified. Key targets, such as MAPK3, STAT3, AKT1, and so on, were pinpointed as central to the network. The identification of these targets suggested that BCBL's therapeutic effect in depression treatment could be due to its influence on multiple critical targets, highlighting a multi‐faceted strategy to tackle the intricacies of depression.

#### 3.2.4 GO and KEGG Enrichment Analysis

To investigate the therapeutic mechanisms of BCBL in depression, we performed a functional analysis of the 192 overlapping targets, focusing on BP, CC, and MF using the R programming language. This analysis revealed 423 significantly enriched GO terms (*p* < 0.05), including 259 BP terms, 62 CC terms, and 102 MF terms. The top 10 enriched terms in each category were graphically displayed in Figure 1D and Figure S3. Significantly, the most prevalent BP terms were protein autophosphorylation, response to drug and, protein phosphorylation, among others. The primary CC terms highlighted integral component of plasma membrane, plasma membrane, and dendrites. In terms of MF, the leading terms were drug binding, protein kinase activity, and protein serine/threonine kinase activity, among others.

Additionally, to explore the signaling pathways through which BCBL might exert its antidepressant‐like effects, we conducted a KEGG enrichment analysis. This analysis identified 113 significant pathways, with the top 10 visualized in Figure 1E and Figure S4. These included the neuroactive ligand‐receptor interaction, calcium signaling pathway, and serotoninergic synapse, among others. The integrated results from the GO and KEGG analyses suggest that BCBL could modulate a broad spectrum of pathways specific to depression, underscoring its potential for a multifaceted approach to treatment.

### 3.3 Effects of Acute Pre‐Treatment With BCBL in Behavioral Despair Mice

**FIGURE 2:**
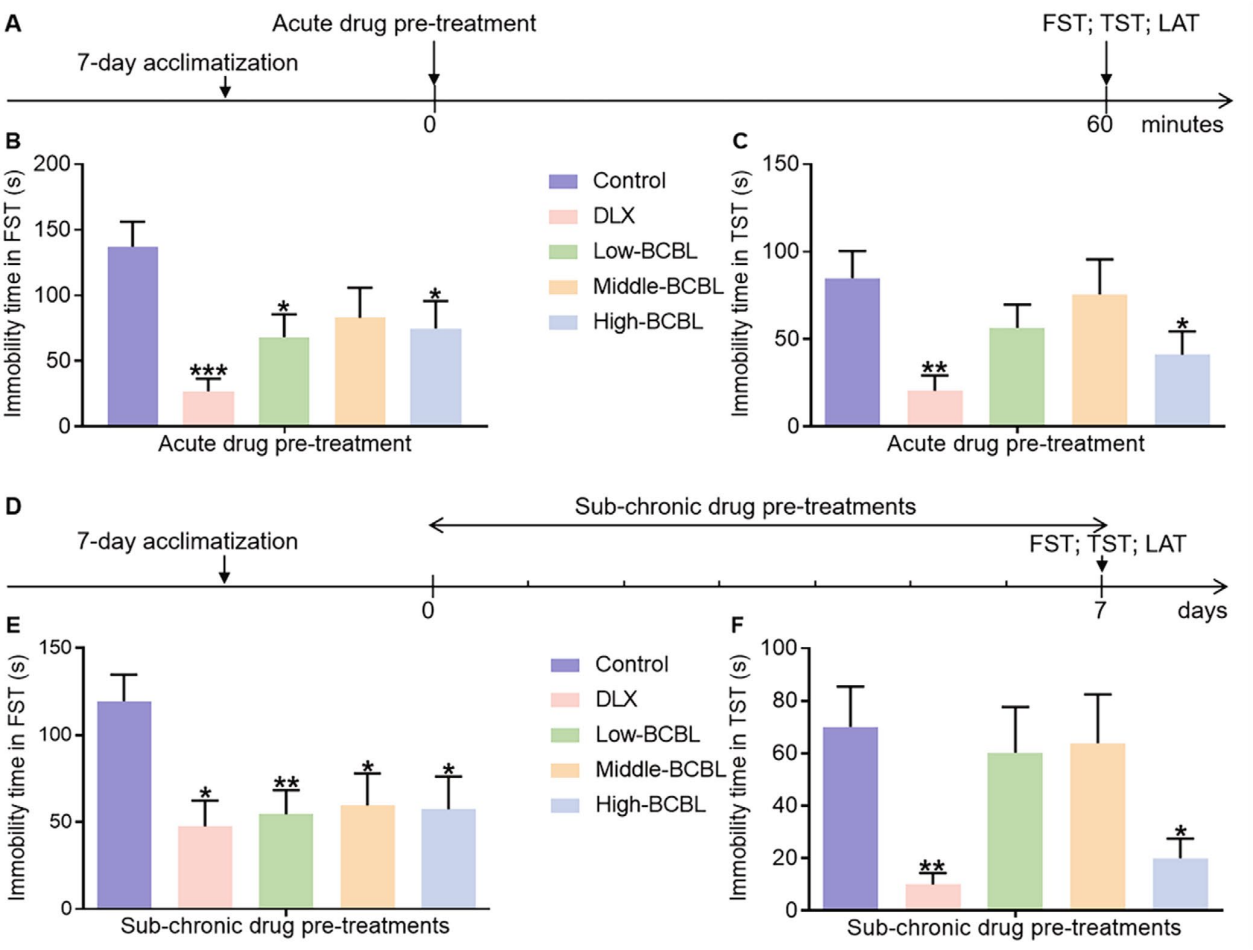
Effects of acute and sub‐chronic pre‐treatments with BCBL in behavioral despair mice. (A, D) Schematic illustrations. (B, C) Effects of acute pre‐treatment with BCBL in FST and TST. (E, F) Effects of sub‐chronic pre‐treatments with BCBL in FST and TST. DLX (20 mg/kg, i.g.) and BCBL at low, middle, and high doses (Water extract & L‐menthol; 300 mg/kg & 1.625 mg/kg, 600 mg/kg & 3.25 mg/kg, and 1200 mg/kg & 6.5 mg/kg, i.g.) were pre‐treated as described in Sections 2.3.2 and 2.4.2. Measurement data were analyzed by Student's *t*‐test or one‐way ANOVA (post hoc LSD test), and presented as mean ± SD (*n* = 10). *p < 0.05, **p < 0.01, ***p < 0.001 in comparison to the control group.

Figure 2A illustrated the initial use of the FST, TST, and LAT to rapidly evaluate the potential antidepressant‐like effects of an acute BCBL pre‐treatment in mice. As depicted in Figure 2B,C, a single dose of DLX (20 mg/kg) significantly reduced the immobility time compared to the control group (*p* < 0.01 and *p* < 0.001, respectively). Post hoc analysis revealed that BCBL, comprising the water extract (1200 mg/kg) and L‐menthol (6.5 mg/kg), also significantly decreased immobility time in the TST (*p* < 0.05). Similarly, BCBL (Water extract & L‐menthol; 300 mg/kg & 1.625 mg/kg, 1200 mg/kg & 6.5 mg/kg) reduced immobility in the FST, achieving statistical significance when compared to the control group (*p* < 0.05). The LAT results, shown in Figure S5A, indicated no significant difference in total distance between the control and drug‐treated groups, thus ruling out false‐positive outcomes due to central stimulation.

### 3.4 Effects of Sub‐Chronic Pre‐Treatments With BCBL in Behavioral Despair Mice

Subsequently, we conducted 7‐day prolonged pre‐treatments with BCBL in mice, followed by behavioral testing to evaluate the sub‐chronic antidepressant‐like effects (Figure 2D–F). The results demonstrated that 7 days of sub‐chronic pre‐treatments with DLX (20 mg/kg) significantly reduced the immobility time in both the FST and TST compared to the control group (*p* < 0.01 and *p* < 0.05, respectively). Further post hoc analysis indicated that sub‐chronic pre‐treatments with BCBL (Water extract & L‐menthol; 1200 mg/kg & 6.5 mg/kg) also significantly decreased immobility time in the TST (*p* < 0.05). Similarly, BCBL (Water extract & L‐menthol; 300 mg/kg & 1.625 mg/kg, 600 mg/kg & 3.25 mg/kg, and 1200 mg/kg & 6.5 mg/kg) led to a significant reduction in immobility duration in the FST (*p* < 0.05 or *p* < 0.01). Analysis of the LAT results, presented in Figure S5B, revealed no significant difference in total distance traveled between the control and drug‐treated groups, indicating no effect on the spontaneous locomotor activity. Collectively, our findings suggested that both acute and prolonged pre‐treatments with BCBL significantly alleviated behavioral despair in mice, indicative of antidepressant‐like effects.

### 3.5 Effects of Chronic Co‐Treatments With BCBL in CRS and CNE‐Induced Depression‐Like Mice

**FIGURE 3:**
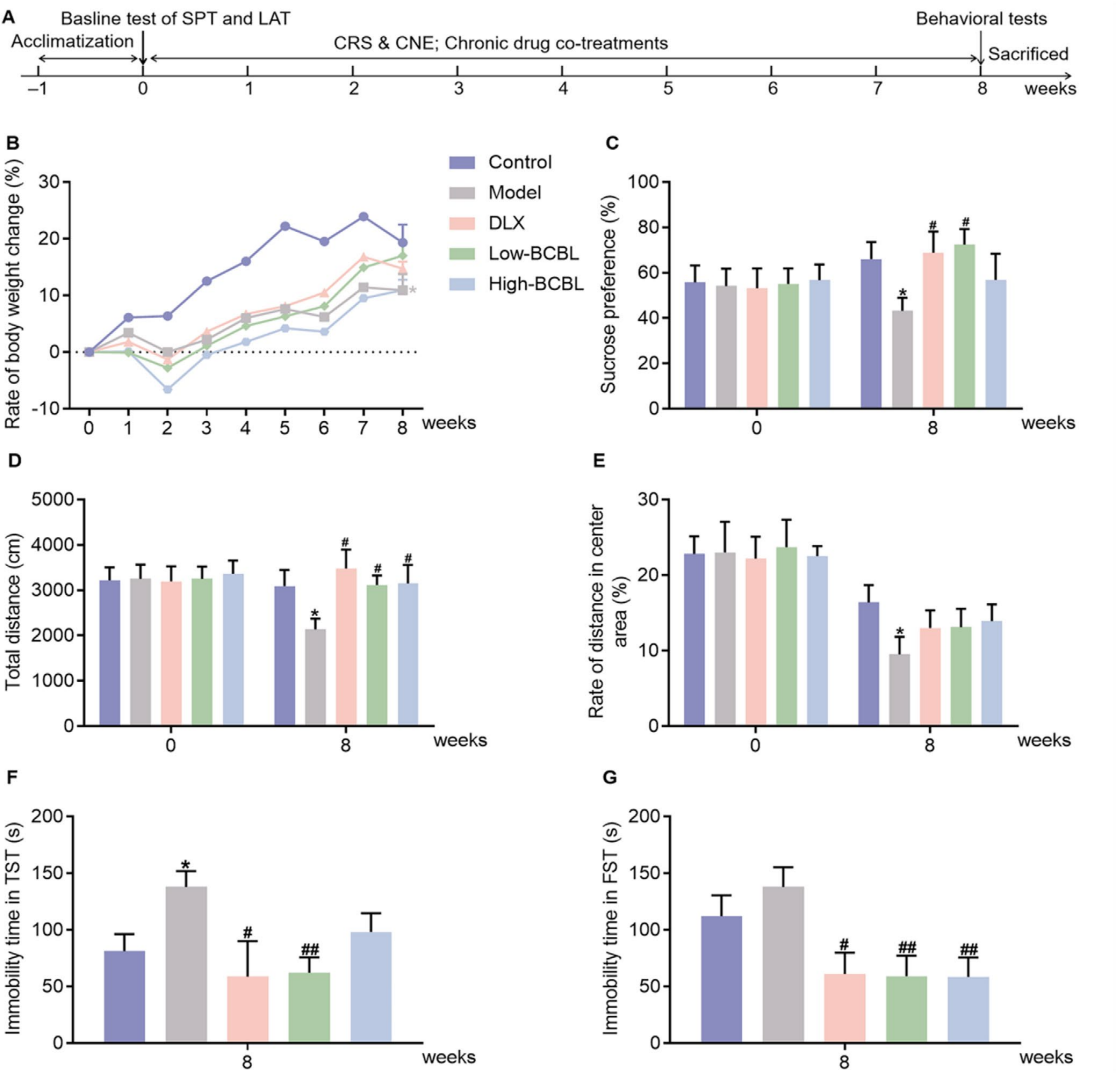
Effects of chronic co‐treatments with BCBL in CRS & CNE‐induced depression‐like mice. (A) Schematic illustration. (B) Rate of body weight change. (C) Sucrose preference index before and after the CRS & CNE procedure along with drug co‐treatments. (D, E) Total distance and rate of distance in the center area before and after the CRS & CNE procedure along with drug co‐treatments. (F, G) Immobility time of CRS & CNE‐induced mice in TST and FST on week 8. DLX (20 mg/kg, i.g.) and BCBL at low and high doses (Water extract & L‐menthol; 1200 mg/kg & 3.25 mg/kg, and 2400 mg/kg & 6.5 mg/kg, i.g.) were co‐treated as described in Section 2.5.2. Measurement data were analyzed by Student's *t*‐test, one‐way ANOVA, or one‐way repeated‐measures ANOVA (post hoc LSD test), and presented as mean ± SD (*n* = 8–10). *p < 0.05 in comparison to the control group; ^#^ *p* < 0.05, ^##^ *p* < 0.01 in comparison to the model group.

Figure 3A illustrated our subsequent investigation using the CRS and CNE‐induced depression model in mice, which was accompanied by comprehensive behavioral testing to assess the potential long‐term antidepressant‐like effects of chronic co‐treatments with BCBL. As shown in Figure 3B, there was no significant difference in the rate of body weight change prior to the CRS and CNE procedure. However, mice subjected to CRS and CNE exhibited a slow increase in body weight by week 8 compared to the control group (*p* < 0.05). Chronic co‐treatments with either DLX (20 mg/kg) or BCBL (Water extract & L‐menthol; 1200 mg/kg & 3.25 mg/kg) could mitigate the slow body weight increase of CRS and CNE‐induced mice to some extent, though no statistical significance was observed (*p* > 0.05).

The SPT was administered to assess the impact of BCBL on anhedonia in CRS and CNE‐induced mice, both before and after the procedure along with drug co‐treatments. Figure 3C showed that there was no significant difference in the sucrose preference index prior to CRS and CNE. However, by week 8, the CRS and CNE procedure significantly reduced the sucrose preference index, indicating anhedonia, compared to the control group (*p* < 0.05). Chronic co‐treatments with DLX (20 mg/kg) significantly increased the sucrose preference index in CRS and CNE‐induced mice by week 8 (*p* < 0.05). Post hoc analysis revealed that BCBL (Water extract & L‐menthol; 1200 mg/kg & 3.25 mg/kg) also significantly ameliorated anhedonia, with a noticeable statistical difference compared to the model group (*p* < 0.05).

To assess the influence of BCBL on the locomotor activity in CRS and CNE‐induced mice, we conducted the LAT both prior to and following the CRS and CNE procedure along with drug co‐treatments. Figure 3D,E showed no significant difference in the locomotor activity before the induction of the model. However, by week 8, the CRS and CNE‐induced mice exhibited a significant reduction in the total distance and rate of distance in the central area compared to the control group (*p* < 0.05). Chronic co‐treatments with DLX (20 mg/kg) effectively reversed the decrease in activity observed in the LAT (*p* < 0.05). Post hoc analysis indicated that BCBL (Water extract & L‐menthol; 1200 mg/kg & 3.25 mg/kg, 2400 mg/kg & 6.5 mg/kg) significantly increased the total distance traveled in the LAT (*p* < 0.05) and partially alleviated anxiety symptoms in the model mice (*p* > 0.05).

The TST and FST were conducted to assess the impact of BCBL on the immobility time in CRS and CNE‐induced mice. Figure 3F,G showed that the CRS and CNE procedure effectively increased the immobility time compared to the control group. Co‐treatments with DLX at 20 mg/kg significantly reduced immobility time in both the TST and FST (*p* < 0.05). Post hoc analysis indicated that BCBL (Water extract & L‐menthol; 1200 mg/kg & 3.25 mg/kg) also significantly decreased immobility time in these tests (*p* < 0.01). Additionally, a significant difference was observed between the BCBL (Water extract+L‐menthol; 2400 + 6.5 mg/kg) group and the model group in the FST (*p* < 0.01).

### 3.6 Effects of Chronic Co‐Treatments With BCBL on the Behavioral Testing in Chronic Reserpine‐Induced Depression‐Like Mice

**FIGURE 4:**
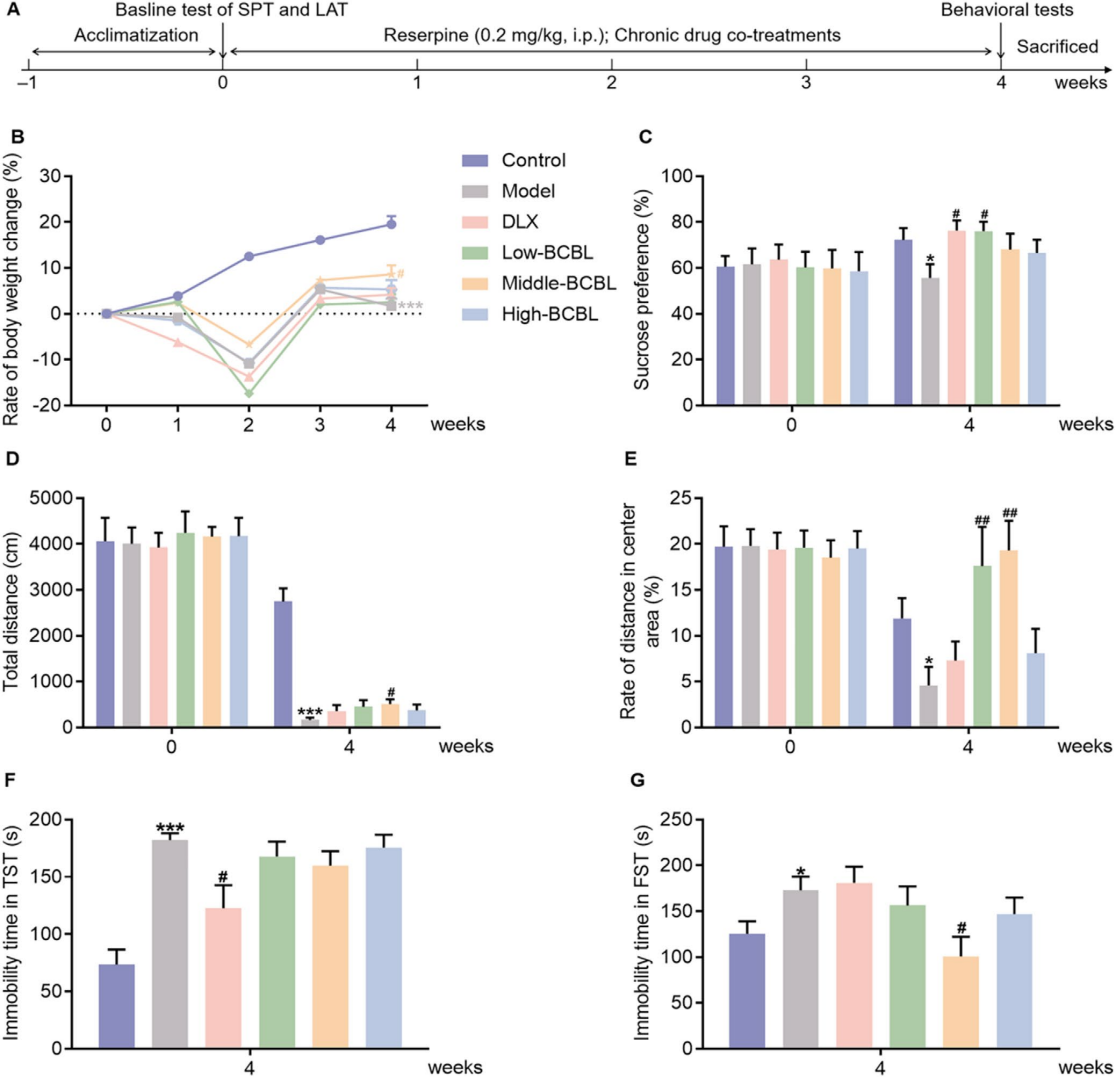
Effects of chronic co‐treatments with BCBL in chronic reserpine‐induced depression‐like mice. (A) Schematic illustration. (B) Rate of body weight change. (C) Sucrose preference index before and after reserpine injections along with drug co‐treatments. (D, E) Total distance and rate of distance in the center area before and after reserpine injections along with drug co‐treatments. (F, G) Immobility time of chronic reserpine‐induced mice in TST and FST on week 4. DLX (20 mg/kg, i.g.) and BCBL at low, middle, and high doses (Water extract & L‐menthol; 600 mg/kg & 1.625 mg/kg, 1200 mg/kg & 3.25 mg/kg, and 2400 mg/kg & 6.5 mg/kg, i.g.) were co‐treated as described in Section 2.6.2. Measurement data were analyzed by Student's *t*‐test, one‐way ANOVA, or one‐way repeated‐measures ANOVA (post hoc LSD test), and presented as mean ± SD (*n* = 11–13). *p < 0.05, ***p < 0.001 in comparison to the control group; ^#^ *p* < 0.05, ^##^ *p* < 0.01 in comparison to the model group.

Figure 4A depicted the establishment of a chronic reserpine‐induced depression‐like model in mice to further assess the potential antidepressant effects of chronic co‐treatments with BCBL. Initial observations, as shown in Figure 4B, indicated no significant difference in body weight prior to drug treatments. However, chronic reserpine injections at a dose of 0.2 mg/kg over a 4‐week period led to a marked slowdown in body weight increase compared to the control group (*p* < 0.001). Post hoc analysis demonstrated that co‐treatments with BCBL, specifically at a dosage of the water extract (1200 mg/kg) combined with L‐menthol (3.25 mg/kg), mitigated the slow body weight increase of chronic reserpine‐induced mice by week 4, with a statistically significant difference observed between the model group and the BCBL‐treated group (*p* < 0.05).

The SPT was administered before and after drug co‐treatments to evaluate the impact of BCBL on anhedonia in chronic reserpine‐induced mice. As depicted in Figure 4C, the sucrose preference index was not significantly different before the modeling process. However, by week 4, chronic reserpine treatments significantly reduced the index, indicating anhedonia, compared to the control group (*p* < 0.05). Chronic co‐treatments with DLX (20 mg/kg) or BCBL (Water extract & L‐menthol; 600 & 1.625, 1200 & 3.25, and 2400 & 6.5 mg/kg, i.g.) could increase the sucrose preference index in the model mice by week 4. Statistically significant differences were observed between both the DLX and the BCBL (Water extract & L‐menthol; 1200 & 3.25 mg/kg) groups and the model group (*p* < 0.05).

As shown in Figure 4D,E, the LAT was conducted to assess the effects of BCBL on the locomotor activity in chronic reserpine‐induced mice. Prior to modeling, no significant difference in the locomotor activity was observed. However, by week 4, chronic reserpine treatments led to a significant reduction in both the total distance traveled and the rate of distance in the central area (*p* < 0.001 and *p* < 0.05, respectively). Co‐treatments with DLX (20 mg/kg) and BCBL (Water extract & L‐menthol; 600 mg/kg & 1.625 mg/kg, 1200 mg/kg & 3.25 mg/kg, and 2400 mg/kg & 6.5 mg/kg) could improve the abnormal activity and alleviate the anxiety symptom in the model mice to varying extents. Notably, the BCBL (Water extract & L‐menthol; 1200 mg/kg & 3.25 mg/kg) group exhibited superior modulatory effects (*p* < 0.05 or *p* < 0.01).

The TST and FST were also processed to determine how BCBL affected the immobility time in chronic reserpine‐induced mice. As shown in Figure 4F,G, chronic reserpine injections significantly elevated these parameters in comparison to the control group (*p* < 0.05 or *p* < 0.001). Co‐treatments with DLX (20 mg/kg) substantially reduced the immobility time in the TST (*p* < 0.05). Post hoc analysis indicated that BCBL (Water extract & L‐menthol; 1200 mg/kg & 3.25 mg/kg) also significantly decreased the immobility time of chronic reserpine‐induced mice in the FST (*p* < 0.05).

### 3.7 Effects of BCBL on the Monoaminergic Systems by DDI Tests

**FIGURE 5:**
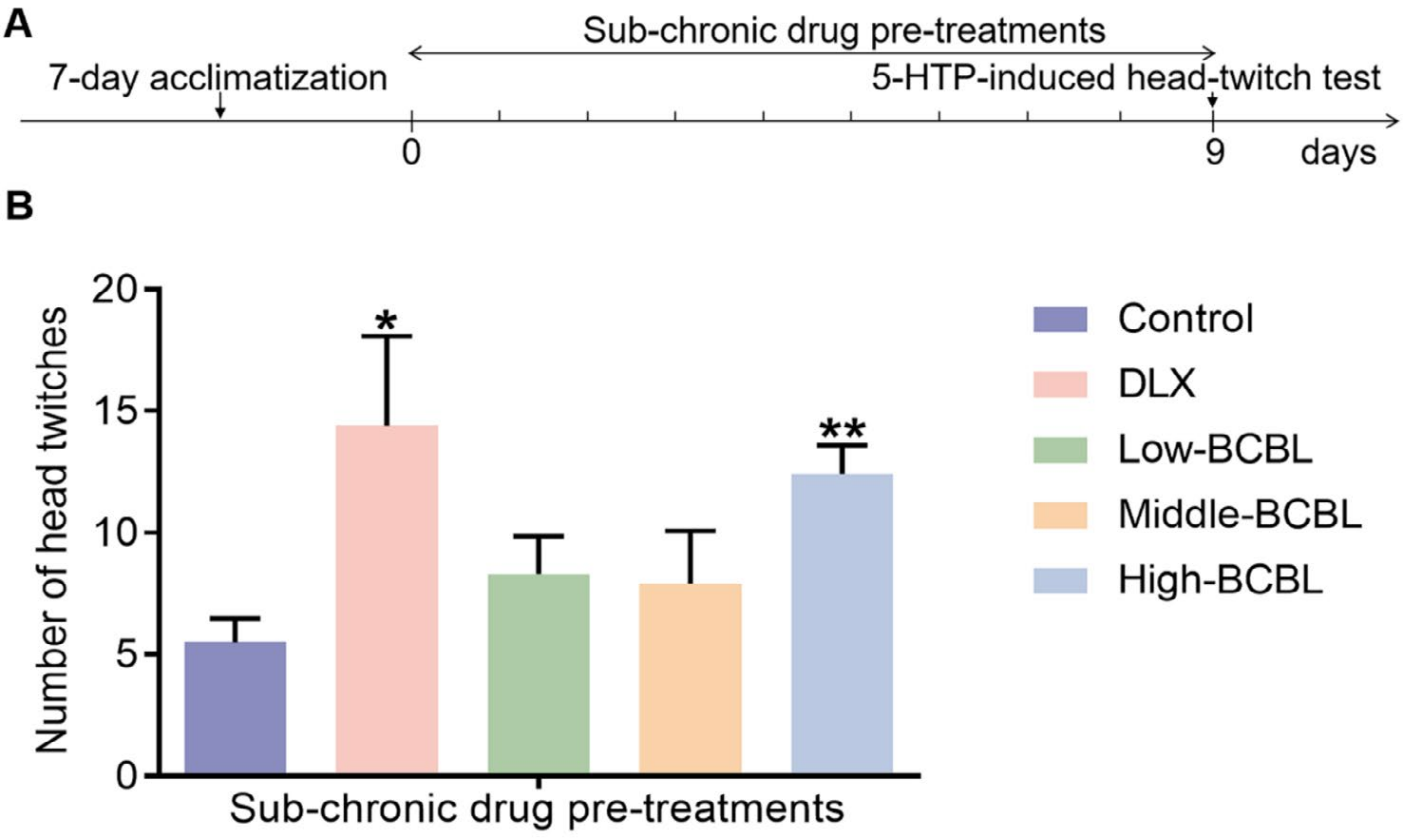
Effects of sub‐chronic pre‐treatments with BCBL on the serotoninergic system by 5‐HTP‐induced head‐twitch test in mice. (A) Schematic illustration. (B) Cumulative number of head twitches during the next 15 min of 5‐HTP‐induced head‐twitch test. DLX (Day 1–8, 20 mg/kg; Day 9, 5 mg/kg, i.g.) and BCBL at low, middle, and high doses (Water extract & L‐menthol; 300 mg/kg & 1.625 mg/kg, 600 mg/kg & 3.25 mg/kg, and 1200 mg/kg & 6.5 mg/kg, i.g.) were pre‐treated as described in Section 2.7.1. Measurement data were analyzed by Student's *t*‐test or one‐way ANOVA (post hoc LSD test), and presented as mean ± SD (*n* = 10). *p < 0.05, **p < 0.01 compared to the control group.

As network pharmacology analysis predicted, the neuroactive ligand‐receptor interaction system, especially the serotoninergic system, might partially mediate the antidepressant‐like effects of BCBL. Thus, the 5‐HTP‐induced head‐twitch test was first utilized to assess the effects of various BCBL doses on the serotoninergic system. The results, as shown in Figure 5, indicated that BCBL significantly enhanced the number of head twitches to varying degrees, with a statistically significant increase observed in the BCBL group (Water extract & L‐menthol; 1200 mg/kg & 6.5 mg/kg) compared to the control group (*p* < 0.01). DLX pre‐treatments, administered at 20 mg/kg from Day 1 to Day 8 and 5 mg/kg on Day 9, also led to a significant increase in head twitches (*p* < 0.05). These findings suggested that the serotoninergic system participated in the antidepressant‐like effects of BCBL.

To evaluate the effects of BCBL on the NEergic system, the yohimbine‐induced death test was carried out after sub‐chronic drug pre‐treatments in mice. As shown in Table 4, DLX (20 mg/kg) increased the number of yohimbine‐induced deaths within 24 h, showing a significant difference compared to the control group (*p* < 0.01). BCBL (Water extract & L‐menthol, 300 mg/kg & 1.625, 600 mg/kg & 3.25, 1200 mg/kg & 6.5 mg/kg) had no significant effect on the number of deaths in mice (*p* > 0.05), suggesting that the NEergic system might not mediate the antidepressant‐like effects of BCBL.

**TABLE 4:** Effects of sub‐chronic pre‐treatments with BCBL on the NEergic system by yohimbine‐induced death test in mice.

Group	Number of death	Total number
Control	2	10
DLX	8**	10
Low‐BCBL	2	10
Middle‐BCBL	3	10
High‐BCBL	3	10

*Note:* DLX (20 mg/kg, i.g.) and BCBL at low, middle, and high doses (Water extract & L‐menthol; 300 mg/kg & 1.625 mg/kg, 600 mg/kg & 3.25 mg/kg, and 1200 mg/kg & 6.5 mg/kg, i.g.) were pre‐treated as described in Section 2.7.2. Count data were analyzed by Fisher's exact test (*n* = 10). **p < 0.01 compared to the control group.

Furthermore, reserpine‐induced ptosis, hypothermia, and akinesia test was conducted to comprehensively evaluate the effects of BCBL on the serotoninergic, NEergic, and DAergic systems. Reserpine, administered at a dose of 2.5 mg/kg, effectively induced ptosis, hypothermia, and akinesia in mice, as indicated in Table 5 (*p* < 0.001). Sub‐chronic pre‐treatments with DLX at 20.0 mg/kg for 9 days significantly mitigated the severity of ptosis and hypothermia (*p* < 0.001 and *p* < 0.05, respectively). BCBL (Water extract & L‐menthol; 300 mg/kg & 1.625 mg/kg, 600 mg/kg & 3.25 mg/kg, and 1200 mg/kg & 6.5 mg/kg) could also ameliorate the degree of ptosis in reserpine‐induced mice. Notably, a significant difference was observed in the degree of ptosis between the BCBL group (Water extract & L‐menthol; 1200 mg/kg & 6.5 mg/kg) and the model group (*p* < 0.05). The results were consistent with the 5‐HTP‐induced head‐twitch test and yohimbine‐induced death test.

**TABLE 5:** Effects of sub‐chronic pre‐treatments with BCBL on the serotoninergic, NEergic, and DAergic systems by reserpine‐induced ptosis, hypothermia, and akinesia test in mice.

Group	Score of ptosis	ΔT/°C	Number of akinesia
Control	0	0.5 ± 0.4	0
Model	3.0 ± 1.3***	2.6 ± 0.6***	8***
DLX	0^###^	2.0 ± 0.6^#^	7
Low‐BCBL	2.6 ± 0.8	2.1 ± 1.0	7
Middle‐BCBL	2.2 ± 0.6	2.6 ± 0.6	8
High‐BCBL	1.6 ± 1.1^#^	2.6 ± 0.7	5

*Note:* DLX (20 mg/kg, i.g.) and BCBL at low, middle, and high doses (Water extract & L‐menthol; 300 mg/kg & 1.625 mg/kg, 600 mg/kg & 3.25 mg/kg, and 1200 mg/kg & 6.5 mg/kg, i.g.) were pre‐treated as described in Section 2.7.3. Count data were analyzed by Fisher's exact test. Measurement data were analyzed by Student's *t*‐test or one‐way ANOVA (post hoc LSD test), and presented as mean ± SD (*n* = 10).

*** *p* < 0.001 compared to the control group.

^#^ *p* < 0.05.

^###^ *p* < 0.001 compared to the model group.

### 3.8 Effects of BCBL on the Serotoninergic, NEergic, and DAergic Systems by HPLC‐ECD

Following the completion of the reserpine‐induced ptosis, hypothermia, and akinesia test, the levels of serotonin, NE, DA, and their respective metabolites 5‐HIAA, DOPAC, and HVA in the hippocampus and PFC were determined by HPLC‐ECD. As shown in Table 6, the levels of serotonin, NE, and DA in the hippocampus of acute reserpine‐induced mice (2.5 mg/kg) were all decreased to varying degrees, with significant differences in serotonin and NE levels compared to the control group (*p* < 0.001). The levels of metabolites 5‐HIAA, DOPAC, and HVA also changed significantly (*p* < 0.01 or *p* < 0.001). Compared with the model group, sub‐chronic pre‐treatments with DLX (20.0 mg/kg) or low and high doses of BCBL (Water extract & L‐menthol; 300 mg/kg & 1.625 mg/kg, 1200 mg/kg & 6.5 mg/kg) for 9 days significantly increased the serotonin level in the hippocampus of model mice (*p* < 0.05 or *p* < 0.01). DLX can also increase the NE level in the hippocampus, though no significant difference was observed compared to the model group (*p* > 0.05).

**TABLE 6:** Effects of sub‐chronic pre‐treatments with BCBL on the levels of serotonin, NE, DA, 5‐HIAA, DOPAC, and HVA in the hippocampus and PFC of mice from reserpine‐induced ptosis, hypothermia, and akinesia test (ng/g tissue).

Group	Serotonin	5‐HIAA	NE	DA	DOPAC	HVA
Hippocampus						
Control	296.8 ± 67.1	924.6 ± 73.8	327.4 ± 62.1	123.7 ± 46.3	201.9 ± 43.6	77.5 ± 19.6
Model	92.9 ± 11.6***	746.5 ± 119.4**	41.5 ± 8.7***	95.4 ± 7.8	91.0 ± 12.0***	146.6 ± 24.8***
DLX	123.7 ± 30.0^#^	532.6 ± 212.9^#^	65.0 ± 46.0	100.9 ± 17.7	74.3 ± 21.5	132.2 ± 35.3
Low‐BCBL	130.9 ± 28.0^##^	906.0 ± 250.9	45.2 ± 8.1	96.7 ± 30.0	93.4 ± 25.1	152.2 ± 32.5
Middle‐BCBL	121.0 ± 40.9	791.8 ± 100.0	40.7 ± 6.0	97.2 ± 13.2	89.5 ± 11.1	148.2 ± 18.4
High‐BCBL	152.6 ± 68.4^#^	838.2 ± 119.4	53.1 ± 26.0	101.4 ± 18.2	84.9 ± 14.7	143.1 ± 34.2
PFC						
Control	366.4 ± 79.2	1012.0 ± 173.1	446.1 ± 104.2	166.6 ± 43.2	248.6 ± 45.0	180.9 ± 22.7
Model	211.4 ± 35.4***	677.3 ± 36.3**	99.0 ± 12.5***	149.0 ± 14.4	227.2 ± 69.9	374.7 ± 107.4***
DLX	225.5 ± 31.6	455.8 ± 125.1^##^	107.0 ± 21.3	151.8 ± 23.8	171.8 ± 43.7	362.5 ± 124.7
Low‐BCBL	210.6 ± 26.5	569.9 ± 144.2	115.8 ± 40.6	136.9 ± 30.7	170.3 ± 48.0	297.7 ± 51.9
Middle‐BCBL	301.3 ± 109.6^#^	756.7 ± 139.7	122.0 ± 31.8	157.6 ± 26.4	206.1 ± 63.0	368.7 ± 114.3
High‐BCBL	262.7 ± 123.4	585.1 ± 239.3	115.8 ± 51.0	137.5 ± 35.0	147.7 ± 57.9^#^	253.4 ± 116.8^#^

*Note:* Measurement data were analyzed by Student's *t*‐test or one‐way ANOVA (post hoc LSD test), and presented as mean ± SD (*n* = 8).

** *p* < 0.01.

*** *p* < 0.001 compared to the control group.

^#^ *p* < 0.05.

^##^ *p* < 0.01 compared to the model group.

The levels of serotonin and NE in the PFC of acute reserpine‐induced mice were also significantly reduced (Table 6; *p* < 0.001), and the levels of metabolites 5‐HIAA and HVA changed significantly (*p* < 0.01 and *p* < 0.001, respectively). Compared with the model group, sub‐chronic pre‐treatments with a middle dose of BCBL (Water extract & L‐menthol; 600 mg/kg & 3.25 mg/kg) for 9 days significantly increased the serotonin level in the PFC of model mice (*p* < 0.05). It was also observed that a high dose of BCBL (Water extract & L‐menthol, 1200 mg/kg & 6.5 mg/kg) could significantly decrease the HVA level in acute reserpine‐induced mice (*p* < 0.05).

### 3.9 Effects of BCBL on the Serotoninergic and NEergic Systems by ELISA

**FIGURE 6:**
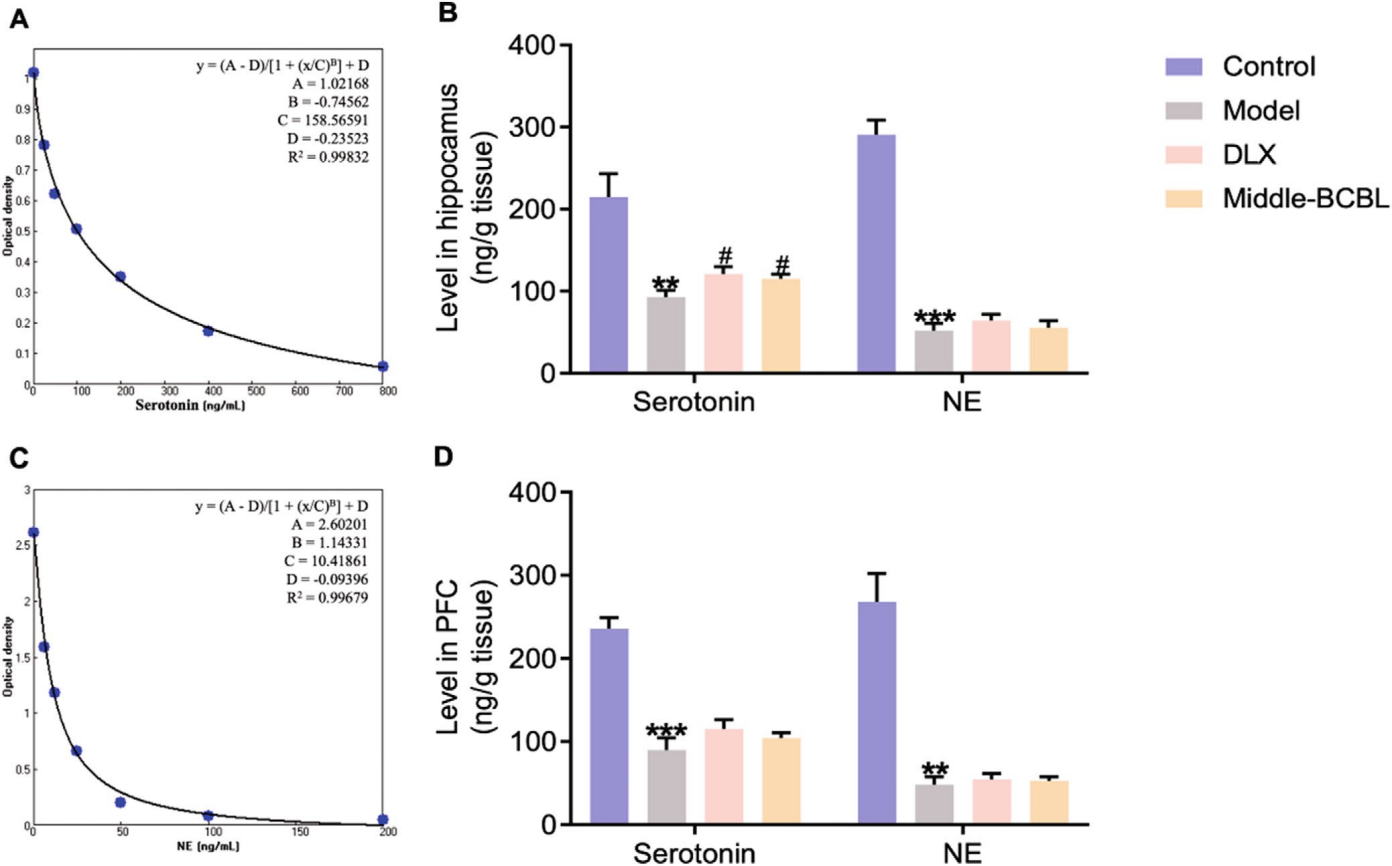
Effects of chronic co‐treatments with BCBL on the levels of serotonin and NE in the hippocampus and PFC of chronic reserpine‐induced depression‐like mice. (A, C) The standard curves and linear regression equations of serotonin and NE by ELISAcalc (Four parameters). (B) The levels of serotonin and NE in the hippocampus. (D) The levels of serotonin and NE in the PFC. Measurement data were analyzed by Student's *t*‐test and presented as mean ± SD (*n* = 6). **p < 0.01, ***p < 0.001 relative to the control group; ^#^ *p* < 0.05 relative to the model group.

The impact of BCBL on the levels of serotonin and NE in the hippocampus and PFC of chronic reserpine‐induced depression‐like mice was further determined by ELISA. As shown in Figure 6B, chronic reserpine injections (0.2 mg/kg) for a period of 4 weeks significantly reduced the levels of serotonin and NE in the hippocampus of model mice (*p* < 0.01 and *p* < 0.001, respectively), while sub‐chronic co‐treatments with DLX (20 mg/kg) or BCBL (Water extract & L‐menthol; 1200 & 3.25 mg/kg) could effectively increase the serotonin level by comparing with the model mice (*p* < 0.05). DLX simultaneously increased the NE level to some extent (*p* > 0.05).

As shown in Figure 6D, it was also observed that chronic reserpine injections (0.2 mg/kg) for a period of 4 weeks significantly reduced the levels of serotonin and NE in the PFC of model mice (*p* < 0.001 and *p* < 0.01, respectively). Sub‐chronic co‐treatments with DLX (20 mg/kg) or BCBL (Water extract & L‐menthol; 1200 mg/kg & 3.25 mg/kg) modestly increased the serotonin and NE levels to some extent, though no significant difference was observed compared to the model group (*p* > 0.05).

## 4 Discussion

The present study comprehensively examined the potential therapeutic effects of BCBL in depression treatment, employing a combination of network pharmacology analysis and experimental validation. First, network pharmacological analysis was conducted to predict the latent antidepressant‐like effects of BCBL. A total of 37 active compounds were identified with the use of the TCMSP database, among which the antidepressant‐like effects of ethyl oleate (Zhang et al. 2021), β‐sitosterol (Yin et al. 2018), stigmasterol (Ghosh et al. 2022), longikaurin A, deoxyharringtonine, angeloylgomisin O, schisandrin B, gomisin A, gomisin G, Gomisin R (Mokhtari and El‐Meghawry El‐Kenawy 2024), and (L)‐menthol (Wang et al. 2019) have been documented in previous studies. Subsequently, a network of these compounds and depression‐related targets was constructed, visualizing interaction between the 37 active compounds and 192 overlapping targets. This network underscored BCBL's role in modulating biochemical pathways associated with depression. PPI network analysis identified core targets such as MAPK3, STAT3, AKT1, PRKACA, MAPK1, and TNF, suggesting BCBL's efficacy in depression treatment may stem from its action on multiple key targets. Additionally, GO analysis revealed 259 BP, 62 CC, and 102 MF terms, while KEGG enrichment analysis pointed to the neuroactive ligand‐receptor interaction, calcium signaling pathway, and serotoninergic synapse as potentially crucial pathways in BCBL's antidepressant‐like effects. These findings from network pharmacological analysis highlighted BCBL's multi‐faceted potential as a promising treatment option for depression.

The FST and TST, two classical behavioral despair tests, along with the LAT, were subsequently conducted to rapidly verify the antidepressant‐like effects of BCBL in mice (Wang et al. 2016). Consistent with prior studies, both acute and sub‐chronic pre‐treatments with DLX (20 mg/kg) successfully led to a decrease in mice's immobility time in the FST and TST without motor stimulation, validating the predictive validity of the behavioral despair depression‐like models (Ciulla et al. 2007; Zhu et al. 2023). Similarly, both acute and sub‐chronic pre‐treatments with BCBL effectively decreased the immobility time of mice in behavioral despair tests. Among these, a high dose of BCBL (Water extract & L‐menthol; 1200 mg/kg & 6.5 mg/kg) displayed superior efficacy in alleviating the desperation of mice. These findings preliminarily substantiate the antidepressant‐like effects of BCBL in typical depression models, suggesting the need for further systematic investigations into its modulatory effects on other depression models and the mechanisms involved.

To further validate the effects of chronic co‐treatments with BCBL, we established a depression‐like model in mice using CRS and CNE. Chronic stress is a well‐known risk factor for the development of depression, and CRS has been widely used to induce depression‐like behaviors in rodents, evaluating the efficacy of potential antidepressants (Qiao et al. 2016; Zhou et al. 2014). Moreover, to address the habituation of rodents to repeated homotypic restraint stressors, we incorporated CNE to enhance the validity of our chronic depression model (Cheng et al. 2022). As a result, the CRS and CNE‐induced mice exhibited characteristics such as a slow increase in body weight, decreased sucrose preference in the SPT, hypolocomotion, and reduced distance in the central area of the LAT. They also showed increased immobility time in the FST and TST, consistent with reported depression features (Cheng et al. 2022). Chronic co‐treatments with DLX (20 mg/kg) successfully ameliorated these symptoms, confirming the good apparent, construct, and predictive validity of the CRS and CNE‐induced depression‐like model. BCBL similarly modulated these behaviors, with the low dose (Water extract & L‐menthol; 1200 mg/kg & 3.25 mg/kg) demonstrating superior efficacy in a comprehensive analysis.

The monoamine depletion hypothesis remains a cornerstone in understanding depression's pathogenesis, guiding the development of first‐line antidepressants like DLX, sertraline hydrochloride, and paroxetine hydrochloride, which are extensively used in clinical practice (Haenisch and Bönisch 2011; Herrman et al. 2022). Reserpine, known to block vesicular monoamine transport, disrupts the serotoninergic, NEergic, and DAergic systems (Henry and Scherman 1989), leading to a chronic depression model in rodents that exhibits emotional and cognitive symptoms along with psychomotor agitation (Abdel‐Rasoul et al. 2023; Qian et al. 2023). Therefore, we utilized the chronic reserpine‐induced depression‐like mice to further verify the potential antidepressant‐like effects of BCBL. Chronic reserpine injections at 0.2 mg/kg over 4 weeks induced a range of depression‐like symptoms in mice, including reduced body weight gain, decreased sucrose preference, hypolocomotion, and increased immobility in despair tests. Co‐treatments with BCBL or DLX effectively reversed these symptoms, confirming the model's predictive validity and BCBL's significant antidepressant‐like effects. Notably, BCBL's effect on body weight gain surpassed that of DLX, aligning with the high remission rates and low toxicity associated with TCM treatments (Zhang et al. 2022; Zhao et al. 2023).

To explore the mechanisms behind BCBL's antidepressant‐like effects, we systematically assessed its impact on monoaminergic function in mice using DDI models, HPLC‐ECD, and ELISA. First, we conducted the 5‐HTP‐induced head‐twitch test and the yohimbine‐induced death test to evaluate BCBL's effects on the serotoninergic and NEergic systems, respectively (Pan et al. 2021; Xu et al. 2017). Subsequently, the reserpine‐induced ptosis, hypothermia, and akinesia test was performed to further evaluate BCBL's effects on the serotoninergic, NEergic, and DAergic systems (Zhu et al. 2023). Our findings showed that DLX significantly increased the number of 5‐HTP‐induced head‐twitch tests, elevated the yohimbine‐induced death rate, mitigated reserpine‐induced ptosis, and counteracted hypothermia. These outcomes aligned with DLX's known action as a dual inhibitor of serotonin and NE reuptake (Engleman et al. 1995). BCBL also effectively enhanced the 5‐HTP‐induced head‐twitch test and alleviated reserpine‐induced ptosis, yet it did not significantly influence reserpine‐induced hypothermia or yohimbine‐induced death. These results suggested that BCBL's antidepressant‐like effects may be mediated primarily through the serotoninergic system.

Following the reserpine‐induced ptosis, hypothermia, and akinesia test, HPLC‐ECD was conducted to accurately determine the levels of serotonin, NE, DA, and related metabolites in the hippocampus and PFC of mice subjected to acute reserpine treatment (Zhang et al. 2012). Our results indicated that both DLX and BCBL could modulate the imbalance of monoamine neurotransmitters and their metabolites caused by an acute reserpine treatment (2.5 mg/kg), particularly by increasing serotonin levels in the hippocampus and PFC. Furthermore, ELISA was conducted to assess the levels of serotonin and NE in the hippocampus and PFC of chronic reserpine‐induced mice (Zhang et al. 2020). The effects of BCBL on the serotoninergic system were consistent with network pharmacology analysis and DDI tests, suggesting that the serotoninergic system might mediate the antidepressant‐like effects of BCBL. This is in line with recent updates on the serotoninergic system in depression (Lin et al. 2023), which highlight the importance of the serotoninergic system in depression treatment.

Moving forward, there are several avenues for future research on the potential use of BCBL in depression treatment. Firstly, employing UPLC‐Q‐TOF‐MS/MS in conjunction with serum pharmacochemistry is essential to identify the chemical constituents of BCBL and clarify its antidepressant material basis. This approach will provide scientific evidence for the modernization of TCM (Gao et al. 2020; Wang et al. 2020). Secondly, it is necessary to evaluate the effects of BCBL on other typical depression models, such as chronic unpredictable mild stress‐induced and chronic social defeat stress‐induced rodents, to further assess its potential as a clinical antidepressant (Antoniuk et al. 2019; Wang et al. 2021). Lastly, given the multi‐component, multi‐target, and multi‐pathway characteristics of TCM, further explorations into aspects like neurotrophic disturbance, abnormal synaptic plasticity, dysregulation of gut microbiota, and so on, will be of great value in elucidating the antidepressant‐like mechanisms of BCBL in depth (Liu et al. 2023; Ménard et al. 2016).

## 5 Conclusion

The present study predicted and validated the antidepressant‐like effects of BCBL, a promising functional food that comprises three safe herbs and a single food additive, including *A. dahurica* , *L. chuanxiong*, *S. chinensis* , and L‐menthol. Through network pharmacology analysis and experimental verification, we have shed light on BCBL's potential in ameliorating depression. The novel formula demonstrated significant modulatory effects in various mouse models of depression, such as behavioral despair mice, CRS and CNE‐induced depression‐like mice, and chronic reserpine‐induced depression‐like mice. Utilizing DDI tests, HPLC‐ECD, and ELISA, we further revealed that the serotoninergic system was involved in the antidepressant‐like effects of BCBL. Our research has uncovered a prospective herbal dietary supplement in depression therapy, and BCBL warrants further investigations and explorations.

## Author Contributions

**Shuai‐Ming Zhu:** conceptualization (equal), writing – original draft (equal). **Chun‐Xue Gao:** formal analysis (equal), investigation (equal). **Zi‐Jia Jin:** data curation (equal). **Fu‐Yao Luo:** methodology (equal). **Ting Feng:** data curation (equal). **Jing‐Cao Li:** Methodology (equal). **Yu Yang:** investigation (equal). **Rui Xu:** validation (equal). **Hao Ma:** software (equal). **Chang‐Wei Li:** supervision (equal), validation (equal). **Jun‐Jie Shan:** supervision (equal), writing – review and editing (equal). **You‐Zhi Zhang:** funding acquisition (equal), project administration (equal), writing – review and editing (equal).

## Conflicts of Interest

The authors declare no conflicts of interest.

## Supporting information


**Figure S1:** Network construction of 37 active compounds and 192 overlapping targets.
**Figure S2:** PPI network analysis involving 192 overlapping targets.
**Figure S3:** GO enrichment analysis.
**Figure S4:** KEGG enrichment analysis.
**Figure S5:** Effects of BCBL on the spontaneous locomotor activity in mice.
**Figure S6:** Chemical structures of serotonin, NE, DA, 5‐HIAA, DOPAC, and HVA.

## Data Availability

Data utilized for supporting research discoveries remains obtainable from the corresponding author as required.
