# Correction to “Baichuan Baile Formula, a Promising Herbal Dietary Supplement, Exerts Antidepressant‐Like Effects by Modulating the Serotoninergic System in Mouse Models”

**DOI:** 10.1002/fsn3.71032

**Published:** 2025-11-27

Zhu SM, Gao CX, Jin ZJ, Luo FY, Feng T, Li JC, Yang Y, Xu R, Ma H, Li CW, Xue R, Shan JJ, Zhang YZ. Baichuan Baile Formula, a Promising Herbal Dietary Supplement, Exerts Antidepressant‐Like Effects by Modulating the Serotoninergic System in Mouse Models. *Food Sci*. *Nutr*. 2025;13(9):e70819. https://doi.org/10.1002/fsn3.70819

In the “Author Contributions” section, the information of the corresponding author, Rui Xue, is missing. The section should be listed as follows:

**Shuai‐Ming Zhu:** conceptualization (equal), writing – original draft (equal). **Chun‐Xue Gao:** formal analysis (equal), investigation (equal). **Zi‐Jia Jin:** data curation (equal). **Fu‐Yao Luo:** methodology (equal). **Ting Feng:** data curation (equal). **Jing‐Cao Li:** methodology (equal). **Yu Yang:** investigation (equal). **Rui Xu:** validation (equal). **Hao Ma:** software (equal). **Chang‐Wei Li:** supervision (equal), validation (equal). **Rui Xue:** methodology (equal), writing – original draft (equal). **Jun‐Jie Shan:** supervision (equal), writing – review and editing (equal). **You‐Zhi Zhang:** funding acquisition (equal), project administration (equal), writing – review and editing (equal).

We apologize for this error.

