# Supplementary material for: Baichuan Baile Formula, a Promising Herbal Dietary Supplement, Exerts Antidepressant‐Like Effects by Modulating the Serotoninergic System in Mouse Models
Source: Food Sci Nutr. 2025 Sep 3;13(9):e70819. doi: 10.1002/fsn3.70819 (PMC12406177; doi:10.1002/fsn3.70819)
Supplement: Supplementary file 1 — Figure S1: Network construction of 37 active compounds and 192 overlapping targets. Figure S2: PPI network analysis involving 192 overlapping targets. Figure S3: GO enrichment analysis. Figure S4: KEGG enrichment analysis. Figure S5: Effects of BCBL on the spontaneous locomotor activity in mice. Figure S6: Chemical structures of serotonin, NE, DA, 5‐HIAA, DOPAC, and HVA. [file FSN3-13-e70819-s001.docx]

**Baichuan Baile formula, a promising herbal dietary supplement, exerts antidepressant-like effects by modulating the serotoninergic system in mouse models**

Shuai-Ming Zhu^a,#^, Chun-Xue Gao^b,a,#^, Zi-Jia Jin^c,a^, Fu-Yao Luo^a^, Ting Feng^d,a^, Jing-Cao Li^a^, Yu Yang^a^, Rui Xu^a^, Hao Ma^a^, Chang-Wei Li^a^, Rui Xue^a,*^, Jun-Jie Shan^a,*^, You-Zhi Zhang^a,*^

^a^ Beijing Institute of Pharmacology and Toxicology, Beijing 100850, China

^b^ North China University of Science and Technology, Tangshan 063210, China

^c^ Beijing Technology and Business University, Beijing 100048, China

^d^ Nanjing University of Chinese Medicine, Nanjing 210046, China

**Contents**

**Fig. S1.** Network construction of 37 active compounds and 192 overlapping targets

**Fig. S2.** PPI network analysis involving 192 overlapping targets

**Fig. S3.** GO enrichment analysis

**Fig. S4.** KEGG enrichment analysis

**Fig. S5.** Effects of BCBL on the spontaneous locomotor activity in mice

**Fig. S6.** Chemical structures of serotonin, NE, DA, 5-HIAA, DOPAC, and HVA

**
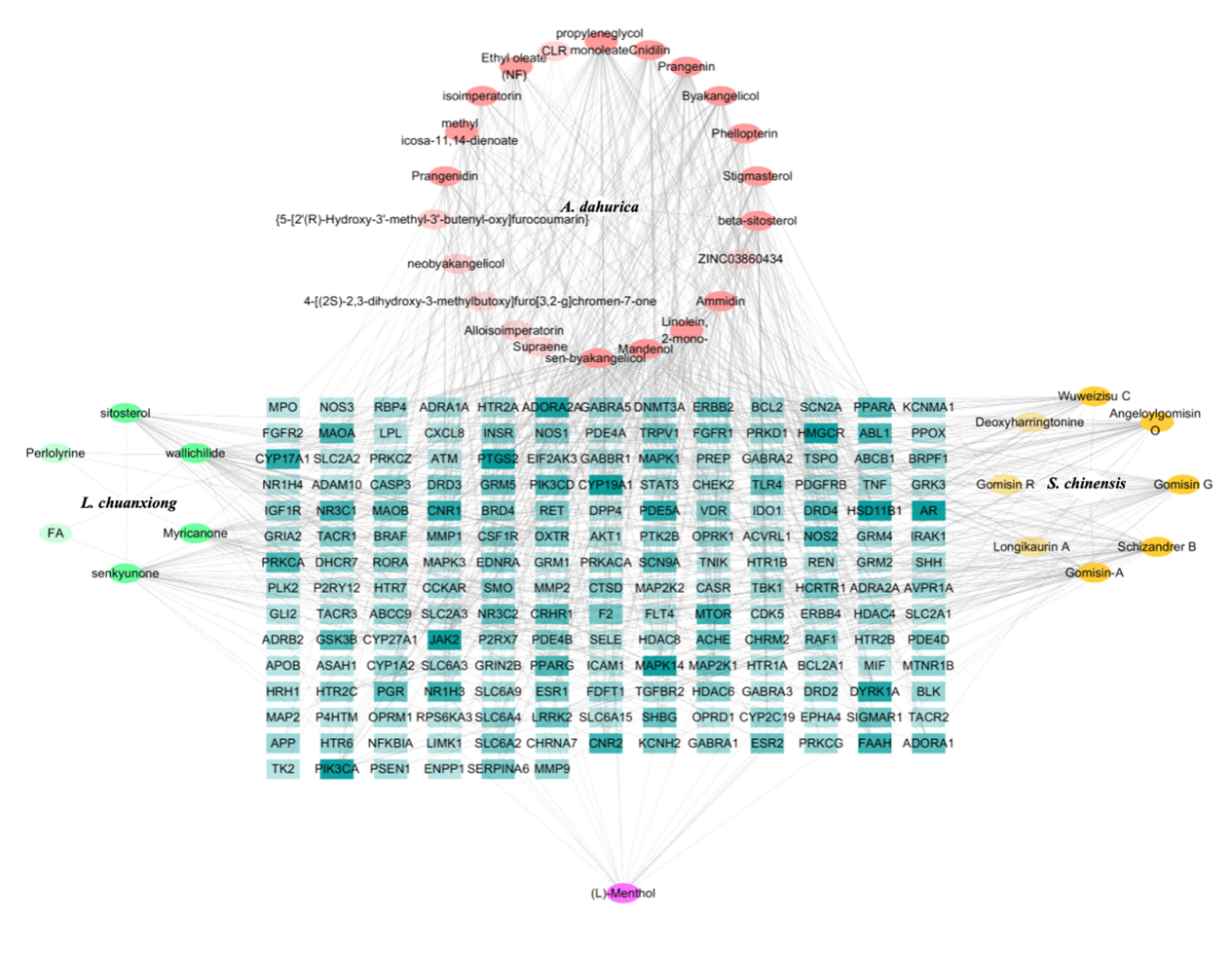
**

**Fig. S1.** Network construction of 37 active compounds and 192 overlapping targets

**
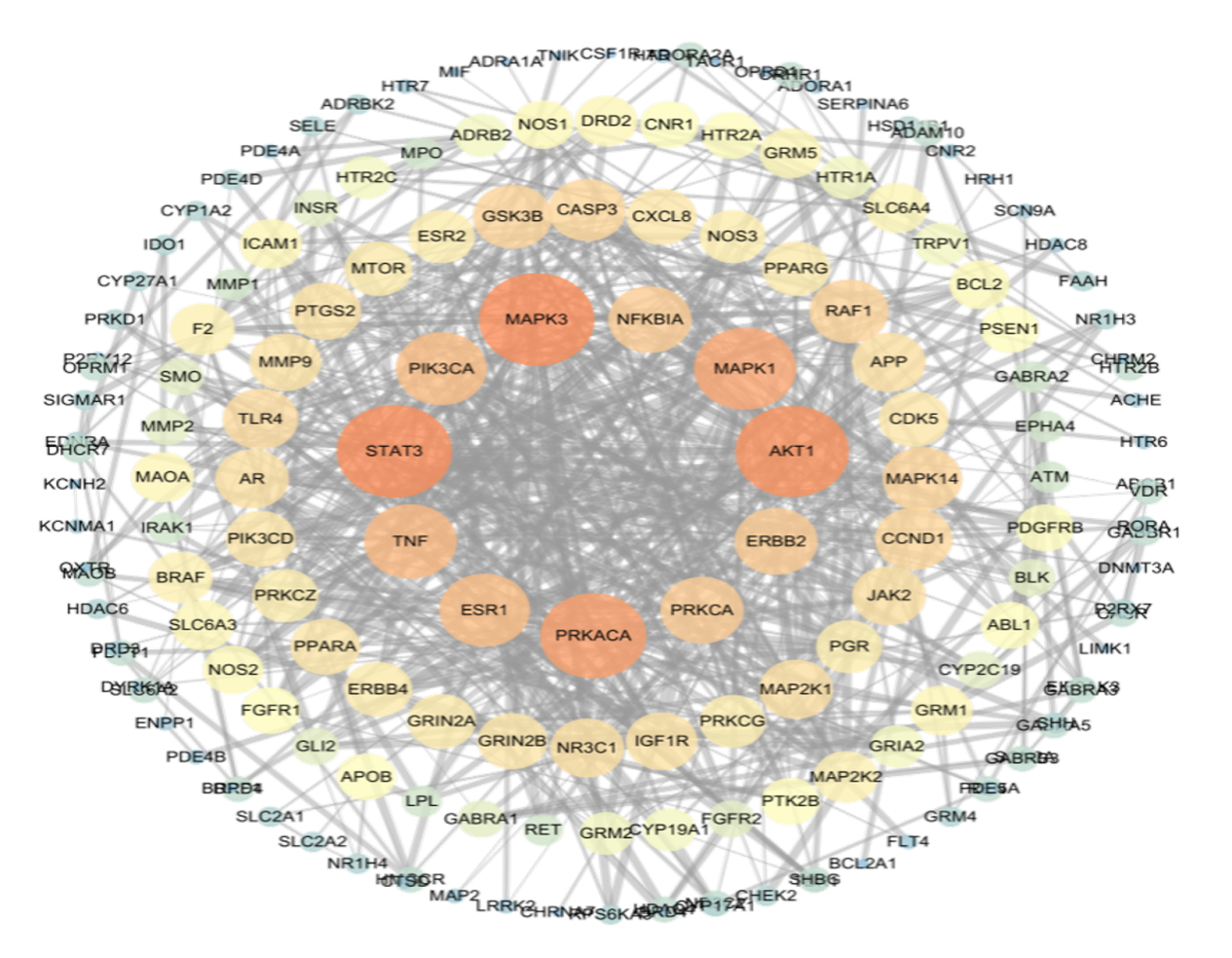
**

**Fig. S2.** PPI network analysis involving 192 overlapping targets

**
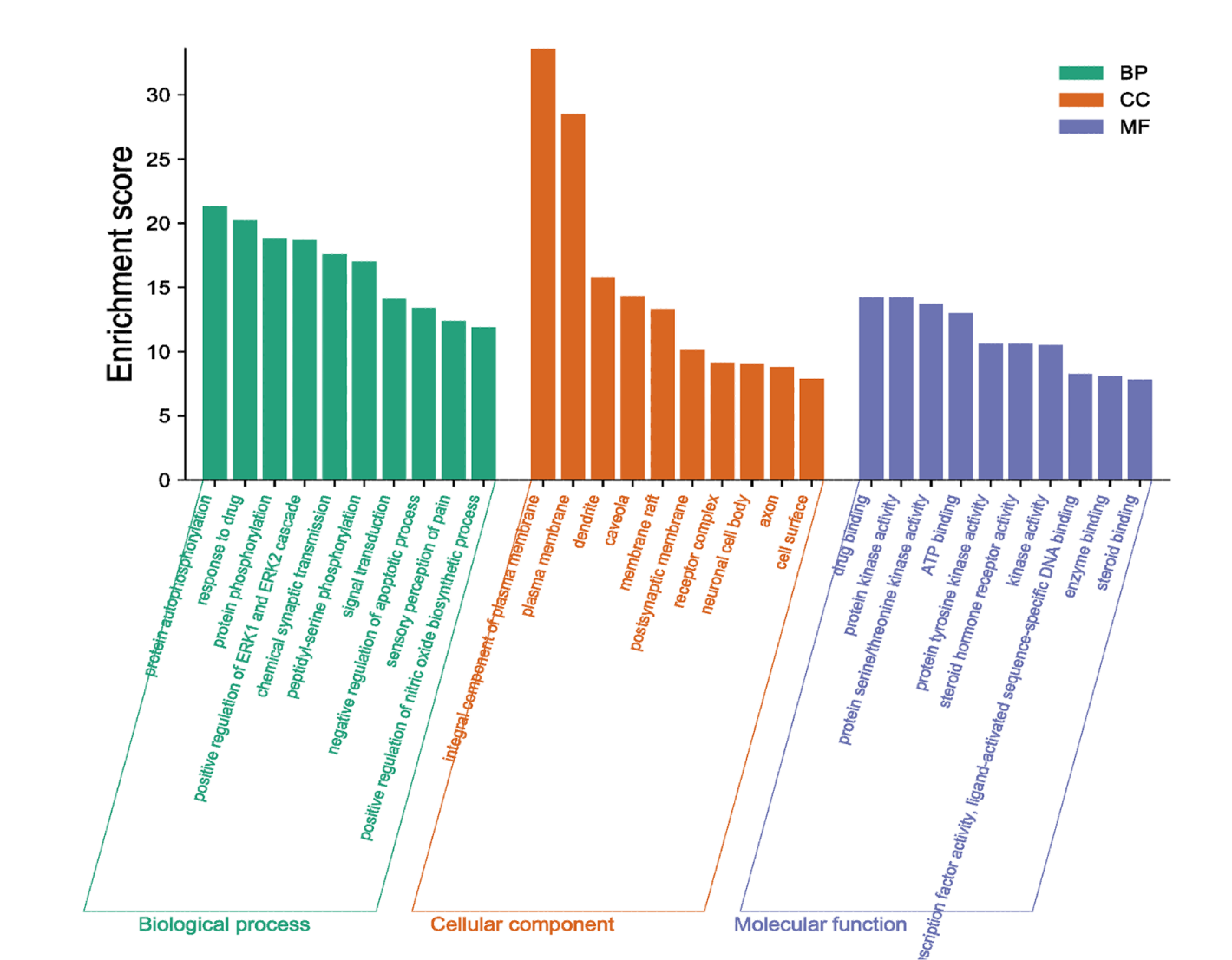
**

**Fig. S3.** GO enrichment analysis


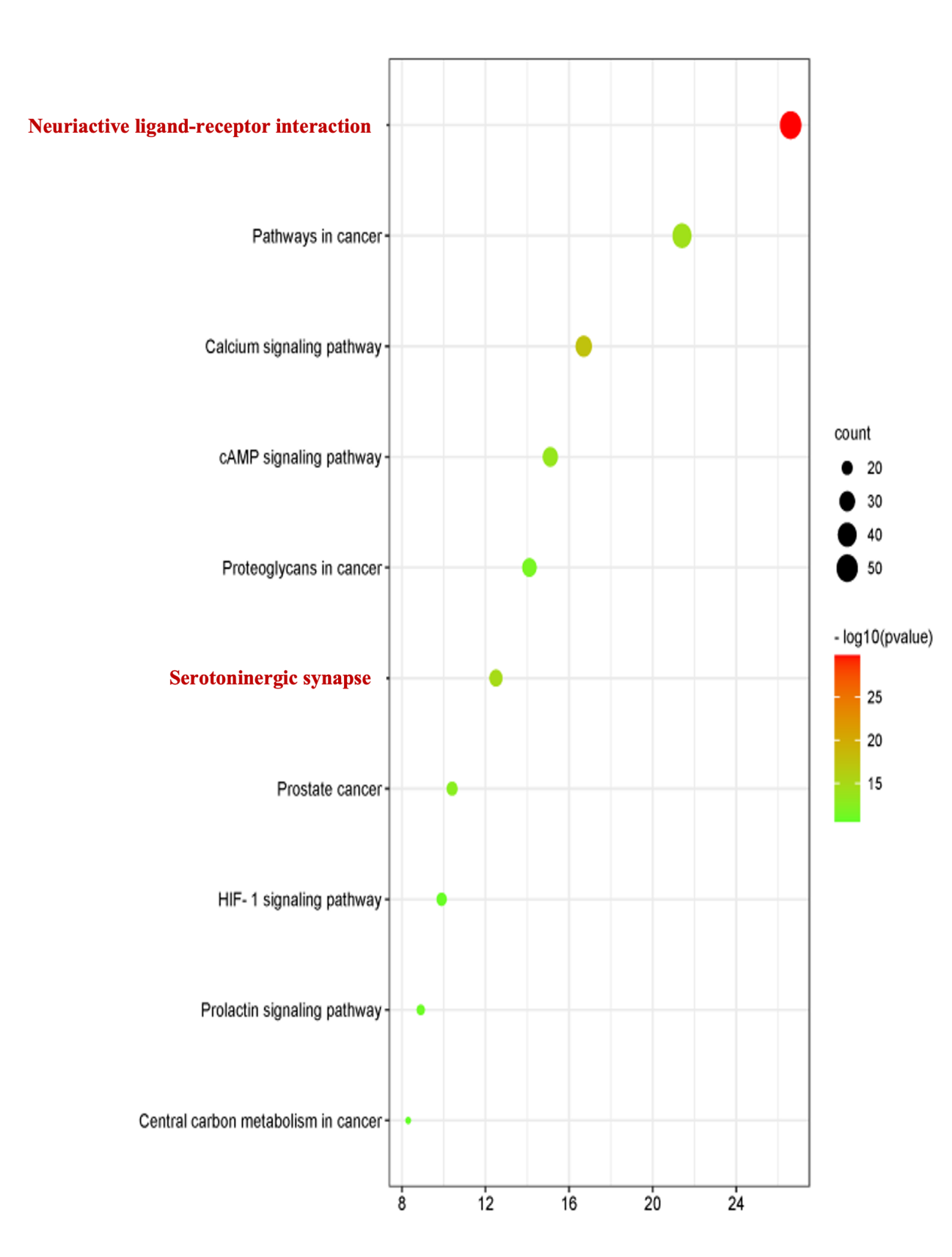


**Fig. S4.** KEGG enrichment analysis

**
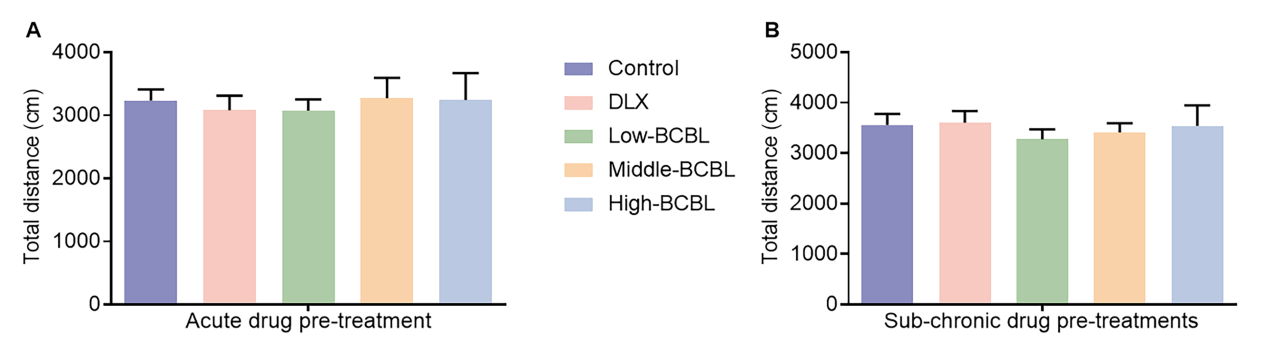
**

**Fig. S5.** Effects of BCBL on the spontaneous locomotor activity in mice. (A) Effects of acute pre-treatment with BCBL on total distance in LAT. (B) Effects of sub-chronic pre-treatments with BCBL on total distance in LAT. DLX (20 mg/kg, i.g.) and BCBL at low, middle, and high doses (Water extract & L-menthol; 300 mg/kg & 1.625 mg/kg, 600 mg/kg & 3.25 mg/kg, and 1200 mg/kg & 6.5 mg/kg, i.g.) were pre-treated as described in sections ***2.3.2*** and ***2.4.2***. Data were presented as mean ± SD (n = 10)

**
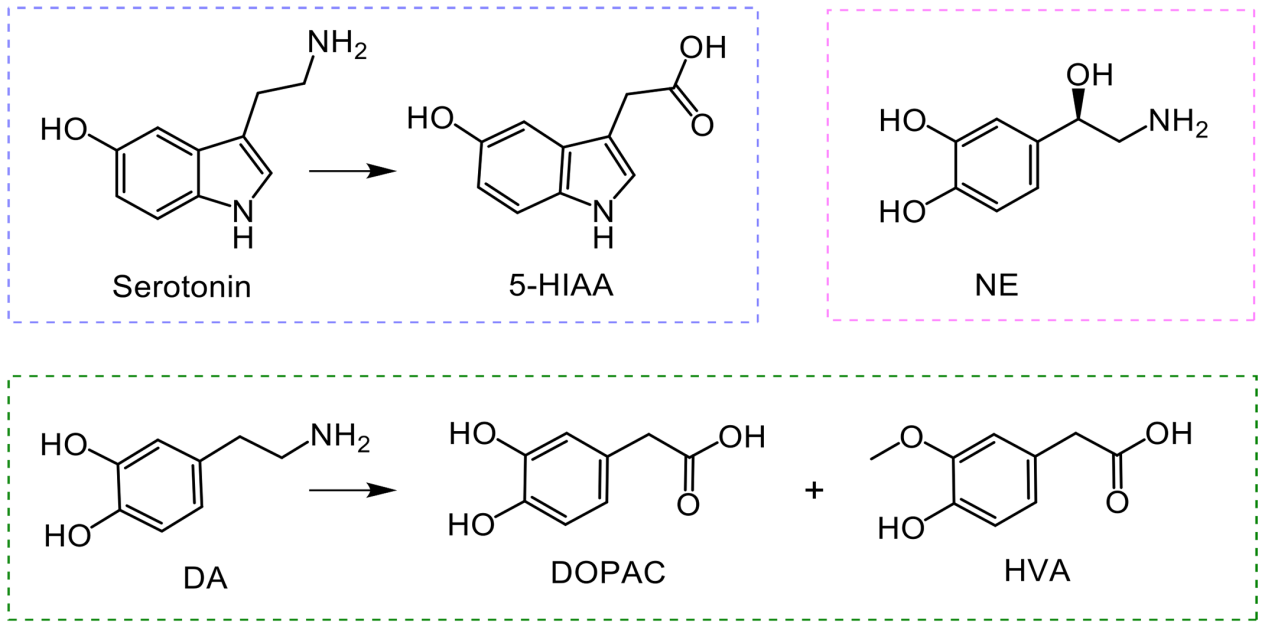
**

**Fig. S6.** Chemical structures of serotonin, NE, DA, 5-HIAA, DOPAC, and HVA
